# An anti-LpqH human monoclonal antibody from an asymptomatic individual mediates protection against *Mycobacterium tuberculosis*

**DOI:** 10.1038/s41541-023-00710-1

**Published:** 2023-08-25

**Authors:** Shivankari Krishnananthasivam, Hao Li, Rania Bouzeyen, Bhuvaneshwari Shunmuganathan, Kiren Purushotorman, Xinlei Liao, Fengjiao Du, Claudia Guldager Kring Friis, Felicity Crawshay-Williams, Low Heng Boon, Qian Xinlei, Conrad En Zuo Chan, Radoslaw Sobota, Mary Kozma, Valeria Barcelli, Guirong Wang, Hairong Huang, Andreas Floto, Pablo Bifani, Babak Javid, Paul A. MacAry

**Affiliations:** 1 Department of Microbiology and Immunology, Yong Loo Lin School of Medicine, National University of Singapore, Singapore, Singapore; 2 College of Veterinary Medicine, China Agricultural University, Beijing, China; 3 Center for Infectious Disease Research, School of Medicine, Tsinghua University, Beijing, China; 4 Division of Experimental Medicine, University of California, San Francisco, USA; 5 Life Sciences Institute, National University of Singapore, Singapore, Singapore; 6 National Clinical Laboratory on Tuberculosis, Beijing Tuberculosis and Thoracic Tumor Institute, Beijing Chest Hospital, Capital Medical University, Beijing, P.R. China; 7 Molecular Immunity Unit, University of Cambridge, Department of Medicine, MRC Laboratory of Molecular Biology, Cambridge, UK; 8 National Centre for Infectious Diseases, Tan Tock Seng Hospital, Singapore, Singapore; 9 Defence Medical and Environmental Research Institute, DSO National Laboratories, Singapore, Singapore; 10 Institute of Molecular and Cell Biology (IMCB), Agency for Science, Technology and Research (A*STAR), Singapore, Singapore

**Keywords:** Biomarkers, Tuberculosis

## Abstract

Tuberculosis (TB) is an airborne disease caused by *Mycobacterium tuberculosis* (Mtb). Whilst a functional role for humoral immunity in Mtb protection remains poorly defined, previous studies have suggested that antibodies can contribute towards host defense. Thus, identifying the critical components in the antibody repertoires from immune, chronically exposed, healthy individuals represents an approach for identifying new determinants for natural protection. In this study, we performed a thorough analysis of the IgG/IgA memory B cell repertoire from occupationally exposed, immune volunteers. We detail the identification and selection of a human monoclonal antibody that exhibits protective activity in vivo and show that it targets a virulence factor LpqH. Intriguingly, protection in both human ex vivo and murine challenge experiments was isotype dependent, with most robust protection being mediated via IgG2 and IgA. These data have important implications for our understanding of natural mucosal immunity for Mtb and highlight a new target for future vaccine development.

## Introduction

Tuberculosis (TB) is an airborne infectious disease caused by the intracellular pathogen *Mycobacterium tuberculosis* (Mtb) that infects humans as a primary host. TB has been a leading cause of death from an infectious disease^1^ and thus a major public health concern. While primary infection with Mtb leads to active disease in around 5–10% of individuals, 90–95% are able to contain the infection in an asymptomatic and non-transmissible state. The emergence of multidrug-resistant (MDR) Mtb strains has become a significant challenge in TB treatment, adding to the global disease burden. This translates into an urgent need for improved TB vaccines where a better understanding of natural immune correlates of protection is vital. Although cell-mediated immunity plays a pivotal role in control of the Mtb infection, the assumption that this is sufficient for vaccine-induced protection is challenged^2–7^, with evidence from multiple cohort studies supporting a role for antibodies in the prevention/control of Mtb infection via mucosal immunity^8–17^. It has also been demonstrated that antibodies interact with extracellular Mtb during the early phase of infection and also with free Mtb antigens present in the granuloma or pleural fluid^18^. IgA is abundantly produced at sites of Mtb infection and the induction of lipoarabinomannan (LAM) secretory IgA upon oral BCG vaccination has been demonstrated^17^. Moreover, previous studies have also shown that intranasal BCG vaccine strategies induce improved protective responses for control of Mtb infection^19^. Monoclonal Mtb surface specific and anti-heparin-binding hemagglutinin (HBHA) IgA antibodies were shown to inhibit bacterial uptake in lung epithelial cells^16^. This study also illustrated a high frequency of IgA anti-HBHA memory B cells in TB-exposed healthcare workers, suggesting that Mtb exposure promotes immunity via IgA responses^16^. A high frequency of IgA memory B cells directed against dormancy-associated antigen Rv2659c was also observed in latent TB infections (LTBI) compared to active TB patients^20^. These data implicate mucosal IgA immune responses as a front line of early host defense for Mtb. By potentially preventing Mtb adherence to mucosal surfaces they support the resolution of infection at the site of initial pathogen entry. Vaccine trials have also shown that antibody responses generated through immunization correlate with reduced risk of active disease^2,6^.

Previous studies conducted on healthy individuals in high Mtb exposure settings have indicated a high degree of antibody-mediated protection with Fc effector functional profiles distinguishing asymptomatic from symptomatic TB patients^10,11,13,14,21^. Characterization of antibody responses in active TB patients have been predominantly associated with secreted and cytosolic antigens rather than surface expressed antigens^22^. In contrast, studies showing protective antibody responses from healthy individuals in high exposure settings principally target Mtb surface expressed antigens^10,11,16,21^. Healthy donors from a high Mtb endemic region developed antibody repertoires against cell membrane antigens such as lipoarabinomannan (LAM) and alpha crystallin (Acr), exhibit antibody-mediated phagocytosis (ADCP) of opsonized Mtb, trigger increased phagolysomal fusion and microbicidal killing activity^10^. Mtb surface capsular arabinomannan (AM) antibodies demonstrated protective efficacy and antibody-mediated phagocytosis, amongst asymptomatic individuals in contrast to active TB patients^21^. These studies implicate surface specific epitopes that potentially allow targeting of extracellular Mtb during the initial stages of infection or help prevent the progression to active disease despite a high exposure to Mtb bacilli.

Investigating IgA/IgG antibody repertoires from asymptomatic individuals in high exposure settings will allow us to gain insights into their specificity and function. Several studies implicate a potential role of IgG2 subclass in protection against Mtb^21,23–25^. These data suggests that Fc engineering of monoclonal antibodies with particular Fc binding characteristics may be crucial for designing new antibody-based prophylactic and therapeutic strategies for TB disease management. In this study, we isolated and thoroughly characterized a human monoclonal antibody represented in the IgG and IgA memory repertoire of a chronically exposed, asymptomatic individual and provide data on specificity, form and function.

## Results

### Donor sample characterization

**Fig. 1:**
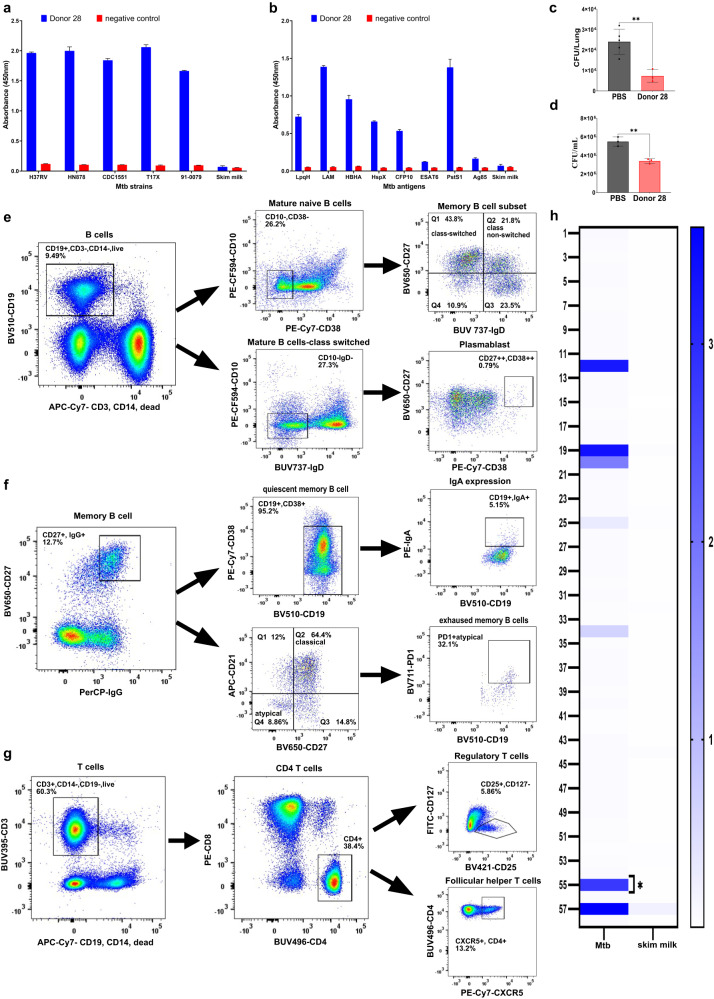
Donor sample characterization and antibody discovery. Purified total Igs of donor 28 was assessed for binding activity against (**a**) Mtb strains and (**b**) common Mtb antigens by indirect ELISA. 3CH5 anti-dengue antibody was used as a negative control. Error bars represent standard error of the mean. **c** In vivo murine protection assay demonstrating significant reduction of lung Mtb bacterial burden in mice administered with 20 mg of total Igs from donor 28. **d** Ex vivo whole blood mycobacterial growth inhibition assay showing significant reduction of Mtb bacterial burden with 50 µg/mL of total Igs from donor 28. Data are expressed as mean ± standard deviation. Statistical significance was determined by two-tailed student t test, *P* < 0.05 is considered as significant. **P < 0.005. **e** Immunoprofiling of B cell subsets showing gating strategy for identification of B cells and further subset population of mature naive B cells, memory B cells and plasmablasts. **f** Immunoprofiling of memory B cells subsets identifying quiescent memory B cells, classical memory B cells, atypical memory B cells and exhausted memory B cells. **g** Immunoprofiling of T cell subsets showing gating strategy for identification of T cells and further subset population of regulatory T cells and follicular helper T cells. **h** Heatmap showing secondary ELISA screening of transfected supernatants-heavy and light chain pairs of donor 28 (Nos-1–56) against whole cell Mtb and skim milk. Untransfected culture supernatant was used as a negative control (No:58), my2F12 anti-LAM antibody spiked into untransfected culture supernatant at 0.5 µg/mL was used as a positive control (No:57).

Total purified Immunoglobulins (Igs) from an occupationally Mtb exposed, asymptomatic healthcare worker donor (no:28) were purified and assessed for binding activity to various Mtb strains and common Mtb antigens. Donor 28 had significant IgG response against five Mtb strains: H37Rv, HN878, CDC1551, T17X, 91-0079 which were representative of four Mtb lineages: Euro-American, East Asian, Indo-Oceanic and East African Indian (Fig. 1a). Donor 28 exhibited significant IgG responses to defined Mtb biomarkers LAM, HBHA, LpqH, HspX, CFP10 and PstS1 (Fig. 1b). The total Igs of donor 28 also demonstrated moderate protection against experimental murine Mtb infection (Fig. 1c) and in an ex vivo human whole blood mycobacterial growth inhibition assay (MGIA) (Fig. 1d) with both showing a significant reduction in mycobacterial burden^11^. A B cell immunoprofile of donor 28 peripheral blood mononuclear cells (PBMCs) was carried out by flow cytometry. B cells were gated by selection of CD19 positive cells and gating out T cells (CD3+) and monocytes (CD14+) (Fig. 1e). CD10, CD27, CD38 and expression of IgD were used to define B cell subsets such as mature naive B cells (CD10-, CD38-), class-switched memory B cells (CD10-, CD27+, IgD-), class-non-switched memory B cells (CD10-, CD27+, IgD+) and plasmablasts (CD10-, IgD-, CD27^high^, CD38^high^) (Fig. 1e). Further characterization of memory B cell subsets was performed using the markers CD27, IgG, PD1 and CD38 identifying quiescent memory B cells (CD27+, IgG+, CD38^low^), classical memory B cells (CD21+, CD27+), atypical memory B cells (CD21-, CD27-) and exhausted memory B cells (CD21-, CD27-, PD1+) (Fig. 1f). Immunoprofiling of T cell subsets was carried out, defining CD4+ T cells (CD3+, CD4+) and gating out B cells (CD19+) and monocytes (CD14+). Markers such as CD25, CD127, CXCR5 were used to identify regulatory T cells (CD25+, CD127-) and follicular helper T cells (CD4+, CXCR5+) (Fig. 1g).

### Human monoclonal antibody discovery

Memory B cells were isolated from donor PBMCs by selection of CD19 positive cells, excluding monocytes and T cells (Fig. 1e), followed by gating for CD27 and IgG or IgA positive cells (Fig. 1f). A final gate to exclude CD38^hi^ B cells was added, to ensure that quiescent memory B cells were obtained by excluding recently generated plasmablasts (Fig. 1f). The IgG positive population is shown as the exemplar (Fig. 1f). From selected memory B cells, IgG positivity was found in 95% and IgA positivity in 5% respectively (Fig. 1f). The selected quiescent memory B cells were then cultured in a cytokine milieu to engender plasma cell differentiation and induce antibody secretion^26^. The B cell culture supernatants were screened for IgG and IgA antibodies that bind to irradiated whole cell Mtb by 384-well, high-throughput ELISA. The ELISA screen indicated several hits of Mtb binding B cell clones with a skew towards IgG (rather than IgA antibodies) (Supplementary Fig. 1). This was as expected since the population of IgA+ memory B cells is smaller (Fig. 1f). The clones with strong binding activity for whole cell Mtb were selected for monoclonal antibody isolation. We identified several heavy and light chain antibody sequences from this analysis, which were then paired into various combinations to identify the original pairings that made up the antibody of interest (Supplementary Table 1). Small scale transfection of the heavy/light chain plasmids was carried out and harvested five days post transfection. Transfected supernatants of heavy/light chain pairs were subjected to a secondary ELISA screen to identify those that show significant binding to Mtb (Fig. 1h). Transfected supernatant no:55 showed strong binding to Mtb, thus identifying a unique monoclonal antibody HuMab-28-009 represented in the IgG and IgA memory repertoire of this donor (Fig. 1h). This antibody was identified to be of IgA isotype in its natural form.

### Antibody engineering

Recent studies on Mtb-specific Fc engineered monoclonal antibodies show effective control of Mtb infection in a neutrophil-dependent manner^24^. A vaccine study showed an increased trend of BCG-specific IgG2 antibody titre in South African infants, being associated with reduced prevalence of Mtb infection^25^. Cohort studies have also shown a pattern of IgG2 responses being associated with mechanisms of protection^21,23^. Therefore, to address if switches in antibody Fc region results in better functional activity, we generated murine IgG1, IgA, human IgG1, IgG2, IgG3 and IgG4 by cloning the variable heavy chain region into the respective IgG subclass or IgA constant region containing plasmids (Fig. 3a). The variable light chain region was cloned into the human lambda constant region containing plasmid (Fig. 3a). Antibody expression was confirmed by SDS-PAGE under reducing conditions, showing bands around 25 and 50 KDa representing the light and heavy chains for all the antibodies, except human IgG3 (Fig. 3b). The human IgG3 shows a band around 70 KDa as it has an elongated hinge region (Fig. 3a, b). Fully assembled antibodies under non-reducing conditions showed a band of around 150 KDa for the murine and human IgG subclasses (Supplementary Fig. 2a). Dimeric murine IgA version of HuMab-28-009 was made to better reflect it’s original isotype. In particular, the dimeric form of this antibody was made to facilitate homing to mucosal sites (including the airways) in the Mtb infection model and assess its protective activity in vivo. A native gel confirmed assembly of the dimeric IgA showing a band above 300 KDa (Supplementary Fig. 2b). A western blot analysis confirmed expression of J chain by probing the immune blot with anti- murine J chain antibody, representing a band above 15 KDa (Supplementary Fig. 2c).

### Antibody binding specificity for mycobacterial species

**Fig. 2:**
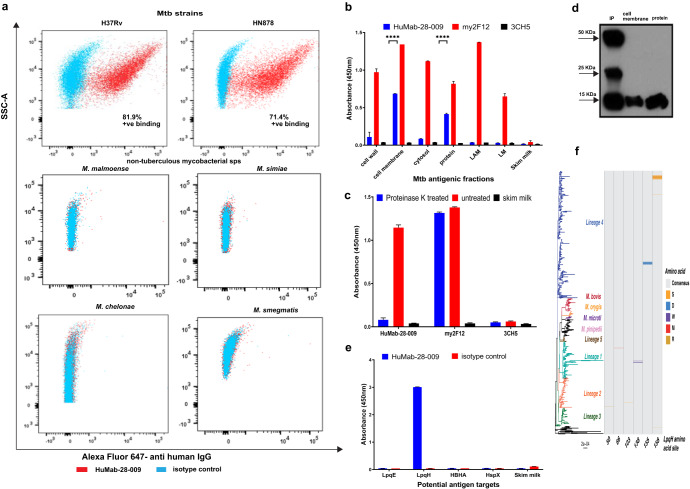
Identification of LpqH as the antigen target of HuMab-28-009. **a** Flow cytometry showing binding activity of HuMab-28-009 to Mtb strains H37Rv, HN878 and no cross reactivity to other mycobacterial species. 3CH5 IgG1 anti-dengue antibody was used as an isotype control. **b** Binding activity of HuMab-28-009 against various antigenic fractions of Mtb, demonstrating significant binding to cell membrane and protein fractions. my2F12 anti-LAM antibody was used as a positive control and 3CH5 anti-dengue antibody was used as a negative control. Error bars represent standard error of the mean. Statistical analysis was done using ordinary one-way ANOVA, with Dunnett’s multiple comparisons test, using GraphPad Prism version 9 and *P* < 0.05 were considered statistically significant. ****P < 0.0001. **c** Proteinase K treatment of Mtb cell membrane fraction followed by indirect ELISA, demonstrates significant reduction of binding activity of HuMab-28-009 against Mtb cell membrane. my2F12 anti-LAM antibody was used as a positive control and 3CH5 anti-dengue antibody was used as a negative control. Error bars represent standard error of the mean. **d** Immune blot analysis of immunoprecipitated (IP) sample, protein and cell membrane fractions probed with HuMab-28-009 identifying the antigen target to be around 15–20 KDa. **e** Indirect ELISA of HuMab-28-009 at a concentration of 5 µg/mL, showing significant binding to LpqH among the potential antigen targets identified by mass spectrometry analysis. Error bars represent standard error of the mean. 3CH5 IgG1 anti-dengue antibody was used as an isotype control. **f** Phylogenetic tree shows the core genome phylogenetic relationship between 630 isolates from across the Mtb complex and is midpoint rooted. Scale bar shows the expected number of nucleotide substitutions per core genome site. Heatmap shows the amino acid at the respective variable position within LpqH relative to the phylogenetic tree. All other 153 sites exhibit a single amino acid across the Mtb complex. Grey shows the consensus (majority) amino acid at the site with variant amino acids shown in different colours (S-Serine in orange, D-Apartic acid in blue, W-Tryptophan in violet, M-Methionine in red, R-Arginine in pale yellow).

The human IgG1 version of HuMab-28-009 was assessed for binding activity to whole-cell mycobacterial species by flow cytometry (gating strategy shown in Supplementary Fig. 5). The antibody showed significant binding to virulent Mtb strains H37Rv and HN878 and no significant cross reactivity to other non-tuberculous mycobacterial species such as *M. malmoense*, *M. simiae*, *M. chelonae* and *M. smegmatis* (Fig. 2a).

### Target antigen identification

The human IgG1 version of HuMab-28-009 was assessed for binding to various Mtb antigenic fractions such as cell wall, cell membrane, cytosol, proteins, LAM and lipomannan (LM). HuMab-28-009 demonstrated significant binding towards the cell membrane and protein fractions of Mtb H37Rv (Fig. 2b). As HuMab-28-009 showed significant binding to the protein fraction, a proteinase K digestion of the Mtb cell membrane fraction was conducted to assess if the binding activity was targeted at a proteinaceous epitope. HuMab-28-009 showed a significant reduction in binding to the cell membrane fraction after proteinase K digestion (Fig. 2c), confirming the proteinaceous nature of the target antigen. The protein fraction of Mtb strain H37Rv was immunoprecipitated with HuMab-28-009. The immunoprecipitated product (IP) was run on SDS-PAGE under reducing conditions, along with Mtb protein and cell membrane fractions. This was followed by probing with HuMab-28-009. The immunoprecipitated product (IP), protein and cell membrane fractions showed a band above 15 KDa which was the likely antigenic target, while bands around 25 and 50 KDa represented the light and heavy chains of the antibody (Fig. 2d). Mass spectrometry analysis of the excised antigenic band from the IP sample revealed four possible antigenic targets: LpqE, LpqH, HBHA and HspX proteins. Binding activity of HuMab-28-009 to the potential antigenic targets was assessed by ELISA, revealing that HuMab-28-009 was specific for LpqH (Fig. 2e). A phylogenetic analysis on the LpqH sequence across 630 Mtb complex isolates (accession nos. included in Supplementary Table 3) was conducted and showed negligible variation, with only 6 mutations observed in the 159 amino acid sequence (Fig. 2f).

### Antibody epitope mapping

An overlapping peptide library, consisting of 15-mer peptides with 10-mer overlap of the LpqH (H37Rv) sequence was used to map the antibody epitope. Dot blotting was performed to scan the peptide library, showing that HuMab-28-009 bound to peptide no:1 with the corresponding sequence VKRGLTVAVAGAAIL (Supplementary Fig. 4). Based upon further comparison of binding activity to additional amino acid sequences, it can be concluded that the antibody-targeted sequence VKRGL (Supplementary Table 2).

### Antibody binding kinetics

**Fig. 3:**
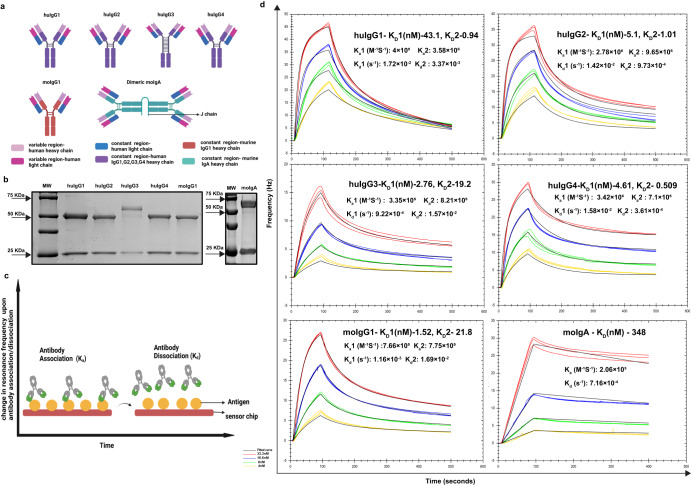
Antibody engineering and biophysical characterization of the chimeric monoclonal antibodies. **a** Illustration of antibody engineering and development of human, murine IgG subclasses and murine IgA dimer. **b** Reducing SDS-PAGE gels showing heavy and light chains confirming antibody expression of the human, murine IgG subclasses and murine IgA. **c** Illustration of the quartz crystal microbalance technology for measuring the antibody binding affinity and kinetics, based on changes in resonance frequency during antigen-antibody interactions in real-time. **d** Binding kinetics (K_a_, K_d_) and affinity (K_D_) of human IgG1, IgG2, IgG3, IgG4 subclass and murine IgG1, IgA dimer antibodies were determined on LpqH coated LNB-carboxyl sensor chip using QCM technology. Antibodies were measured in triplicates at varying concentrations. 1:2 binding model was the best curve fitting for the IgG antibodies, while 1:1 binding model was the best fit for the IgA antibody. TraceDrawer software was used for analysis. Figures 3a and c were created using BioRender.com.

The binding activity of the IgG subclass and IgA antibodies to recombinant LpqH was assessed by ELISA (Supplementary Fig. 3). The human IgG3 subclass exhibited stronger binding activity compared to the other human IgG antibodies (Supplementary Fig. 3a). Binding kinetics and affinity of the human IgG subclass and murine IgG1, IgA dimer antibodies were assessed by Quartz Crystal Microbalance (QCM) technology. This allowed for a real-time determination of binding association rate (K_a_), dissociation rate (K_d_) and binding affinity (K_D_). Binding kinetics and affinity were analysed and curve fitting performed using TraceDrawer software (Ridgeview Instruments). The IgG antibodies displayed bivalent interactions (1:2 binding model) with strong binding affinities (K_D_) in the nM range and no significant variation between subclasses (Fig. 3d, Table 1). Human IgG2, IgG3 and IgG4 antibodies displayed very slow dissociation rates (Fig. 3d, Table 1). A 1:1 binding model was the best fit to analyse the murine IgA antibody, demonstrating very slow dissociation rates (K_d_) (Fig. 3d, Table 1).

**Table 1:** Binding affinity and kinetics determination of human, murine IgG subclasses and murine IgA isotype of HuMab-28-009.

Antibody subclass	K_a_1 (M^–1^S^–1^)	K_d_1 (s^–1^)	K_a_2 (M^–1^S^–1^)	K_d_2 (s^–1^)	K_D_1 (nM)	K_D_2 (nM)
Human IgG1	4 × 10^5^	1.72 × 10^–2^	3.58 × 10^6^	3.37 × 10^–3^	43.1	0.94
Human IgG2	2.78 × 10^6^	1.42 × 10^–2^	9.65 × 10^5^	9.73 × 10^–4^	5.1	1.01
Human IgG3	3.35 × 10^5^	9.22 × 10^–4^	8.21 × 10^5^	1.57 × 10^–2^	2.76	19.2
Human IgG4	3.42 × 10^6^	1.58 × 10^–2^	7.1 × 10^5^	3.61 × 10^–4^	4.61	0.509
Murine IgG1	7.66 × 10^5^	1.16 × 10^–3^	7.75 × 10^5^	1.69 × 10^–2^	1.52	21.8
Murine IgA	2.06 × 10^3^	7.16 × 10^–4^			348	

Binding kinetics (K_a_, K_d_) and affinity (K_D_) of antibodies assessed by Quartz crystal microbalance (QCM) technology, with binding affinity in nM range.

### Protective activity of HuMab-28-009 against experimental Mtb infection

**Fig. 4:**
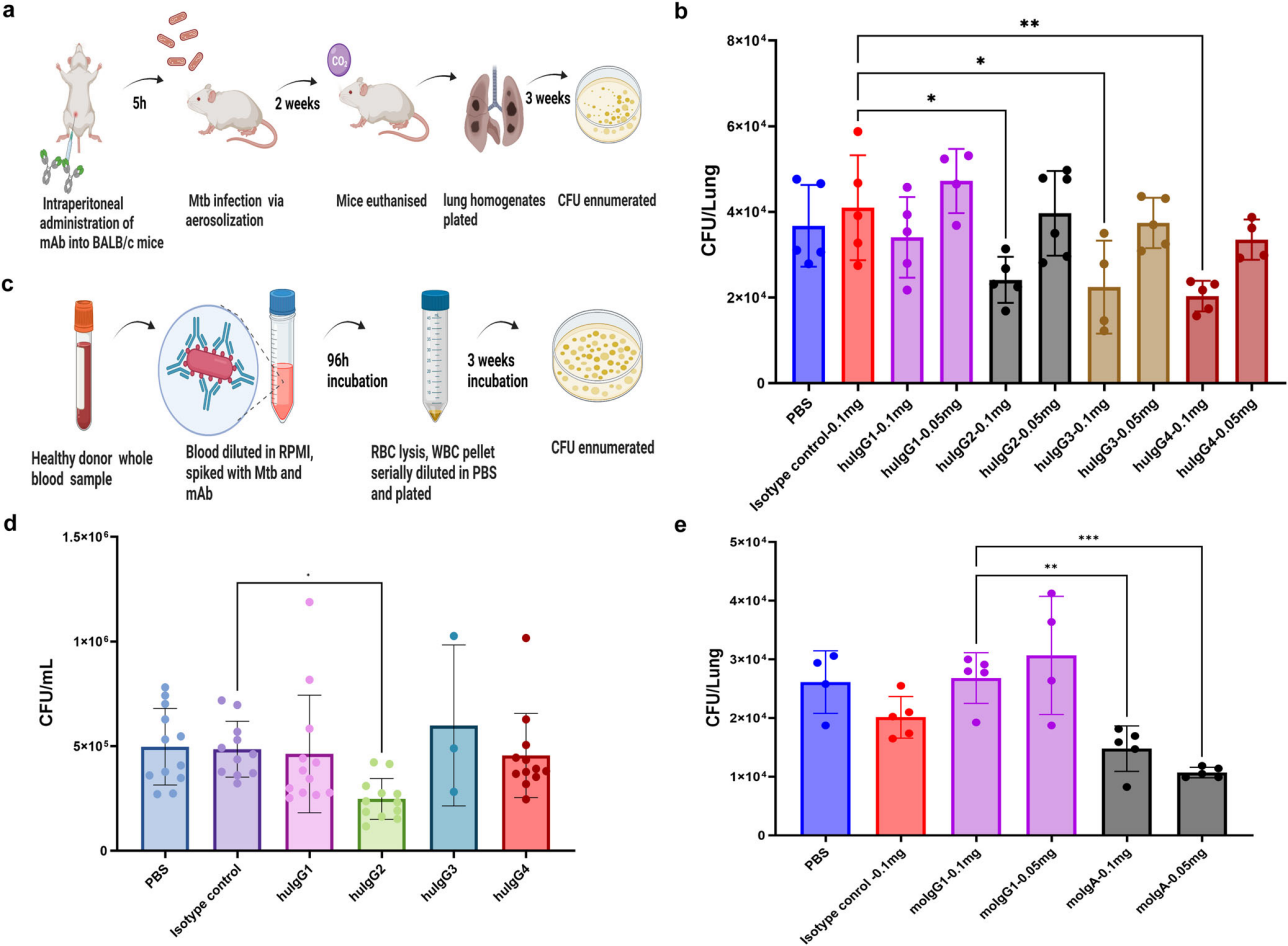
Determination of protective activity of the chimeric monoclonal antibodies against Mtb. **a** Illustration of the murine protection assay. **b** Murine protection assay shows that human IgG2, IgG3 and IgG4 subclasses at a dose of 0.1 mg per mouse demonstrates significant reduction of Mtb bacterial burden compared to non-specific IgG1 isotype control at a dose of 0.1 mg per mouse. **c** Illustration of the whole blood mycobacterial growth inhibition assay (MGIA). **d** Whole blood MGIA reveals that human IgG2 subclass demonstrates significant Mtb growth restriction compared to non-specific IgG1 isotype control. Four independent experiments were done in triplicates for all antibodies except human IgG3. **e** Murine protection assay shows that murine IgA dimer in both doses demonstrates significant reduction of Mtb bacterial burden compared to murine IgG1 antibody at a dose of 0.1 mg per mouse. Data are expressed as mean ± standard deviation for the in vivo, ex vivo assays. Statistical significance was determined by one-way ANOVA, with Dunnett’s multiple comparisons test, where *P* < 0.05 is considered as significant. *P < 0.05, **P < 0.005 and ***P < 0.001. Figures 4a and c were created using BioRender.com.

We assessed IgG subclasses and dimeric IgA of HuMab-28-009 for protection against aerosol infection of mice with Mtb. Two doses (0.05 and 0.1 mg/mouse) of monoclonal antibody were injected intraperitoneally to BALB/c mice 5 h prior to aerosol infection with the lineage 2 Mtb strain HN878. Bacterial burden in the lungs was assessed 14 days later (Fig. 4a). Human IgG2, IgG3 and IgG4 antibodies, but not human IgG1 at a dose of 0.1 mg per mouse demonstrated significant reduction of Mtb in murine lungs compared with a non-specific human IgG1 isotype control at a dose of 0.1 mg per mouse (Fig. 4b). In our assessment of murine IgG1 and IgA antibodies, the dimeric murine IgA in both doses showed a significant reduction of Mtb burden compared to murine IgG1 at a dose of 0.1 mg per mouse (Fig. 4e). When testing the human IgG subclasses in an ex vivo human whole blood mycobacterial growth inhibition assay (MGIA) using four distinct donors, only the IgG2 subclass offered protection (Fig. 4d). The human IgG2 showed a significant Mtb growth restriction compared to human IgG1 isotype control (Fig. 4d).

## Discussion

This study describes the isolation of a novel IgA monoclonal antibody directly from human samples by screening single-activated memory B cells. The characterization of natural antibodies isolated from Mtb exposed asymptomatic individuals represents a valuable resource for identifying new targets for Mtb prophylaxis. This study compliments related analyses where human recombinant monoclonal antibodies against Mtb have been isolated from the memory B cells of TB patients or asymptomatic TB healthcare workers^3,16,27^.

An asymptomatic healthcare worker (donor 28) exhibited a significant IgG response to immunological biomarkers LAM, HBHA, LpqH, HspX, CFP10 and PstS1 (Fig. 1b). Strong IgG and IgA responses to ESAT6/CFP10, LAM, LpqH and HspX antigens have been observed in LTBI individuals^28–32^. Donor 28 despite being negative by interferon-gamma release assay (IGRA) to ESAT6/CFP10 antigens, showed a moderate IgG response to CFP10 antigen (Fig. 1b). A cohort study demonstrated that asymptomatic individuals who were IGRA negative, nonetheless had detectable non-IFNγ T cell response and persistent IgG responses to Mtb antigens E-SAT6 and CFP10, suggestive of IFNγ-independent immune mechanisms and class-switched humoral immunity in mediating protection^14^. Likewise, significant antibody responses against Mtb antigens HspX, GlcB, MPT51 have been identified in tuberculin skin test (TST) negative asymptomatic healthcare workers, suggesting a potential protective role for humoral immune responses independent of cellular response mechanisms^33^. The total Igs of donor 28 also demonstrated significant protective response against Mtb both by ex vivo MGIA and aerosol murine infection (Fig. 1c, d). Thus, this protective donor was selected to identify fully human monoclonal antibodies, that could mediate protection in experimental Mtb infection.

The employment of whole cell Mtb as opposed to discrete antigens as ‘bait’ was based on findings from previous published studies demonstrating that protective antibody responses from asymptomatic healthcare workers target surface expressed antigens^10,11,16,21^. Our strategy represents an unbiased holistic approach by which potential protective antibodies can be identified. We observed strongly binding IgA and IgG antibodies from our memory B cell screening process (Supplementary Fig. 1). Uniquely, our antibody screening process revealed a monoclonal antibody HuMab-28-009, which existed as the IgA1 subclass with a lambda light chain in its natural form, indicative of its mucosal origin. The generation of this type of antibody is predicated on natural exposure to Mtb. We chose to focus on HuMab-28-009, as it was represented in the IgA repertoire and evaluated whether it mediated protection in experimental Mtb infection.

The antibody was expressed as a human IgG1 for initial characterization, while human IgG2, IgG3, IgG4 and murine IgG1, IgA were made to ascertain if the binding affinity and protective responses against Mtb could be improved (Figs. 3, 4). The antibodies showed significant binding to Mtb strains H37Rv and HN878, while showing no significant cross-reactivity to non-tuberculous mycobacterial species (Fig. 2a). We went on to identify the antigenic target of HuMab-28-009 as LpqH (Fig. 2e), which has been implicated in host evasion and maintenance of chronic infection. LpqH is a 19 KDa cell membrane anchored lipoprotein, implicated in TLR2-dependent inhibition of MHC class II expression and antigen processing^34–36^. LpqH has been shown to inhibit IFNγ dependent histone acetylation at the promoter locus (pIV) of MHC class II transactivator (CIITA) gene, via TLR2-induced MAPK signalling, thus leading to suppression of CIITA transcription^37^. Prolonged stimulation of TLR-2 by LpqH, inhibited IFNγ-regulated MHC class II mRNA and protein expression in macrophages, thus leading to downregulated MHC class II-dependent antigen presentation and CD4 T cell responses^34^. A comprehensive study into the proteoform repertoire of LpqH indicated complex post-translational modifications such as acylations, glycosylations, phosphorylations and some truncations^38^. The Mtb LpqH gene shares homology within the Mtb complex species, with our phylogenetic analysis across 630 isolates showing low levels of variability, with only 6 mutations observed among the 159 amino acid sequence (Fig. 2f). However variability in protein expression and post translation modifications in Mtb could play a major role in its pleotropic biological activities and thereby contribute to virulence and pathogenicity of Mtb^39^.

The identification of the antibody epitope of LpqH, which is targeted by the natural mucosal immune system of an asymptomatic individual should be beneficial for future vaccine strategies. From peptide library scanning we identified that the antibody targets the N terminal portion of the protein (Supplementary Fig. 4, Supplementary Table 2). Further structural studies are needed to fully understand the epitope-paratope interactions.

Biophysical assessment of the IgG and IgA chimeric monoclonal antibodies by QCM revealed bivalent interaction among the IgG subclass with a high binding affinity in nM range (Fig. 3d, Table 1). Human IgG2, IgG3, IgG4 and murine IgA antibodies displayed very slow dissociation rates (K_d_) (Fig. 3d, Table 1), indicating a stable and longer antigen-antibody interaction, which may offer better protection. This antibody exists as an IgA1 in its natural form. On comparison with top matching germline IGHV4-39*01, the heavy chain had seven amino acid mutations as a result of fourteen nucleotide changes (Table 2). The light chain, on comparison to its top germline match IGLV2-11*01 had six amino acid mutations resulting from six nucleotide changes (Table 2). These observations indicate that the HuMab-28-009 had undergone some, but not extensive affinity maturation, where typically in each round of affinity maturation, 1–2 nucleotides in the heavy or light chain sequence are mutated^40^.

**Table 2:** CDR sequences of isolated antibody HuMab-28-009.

Antibody	CDR1	CDR2	CDR3	Germline
Light chain	S S D V G **R** Y N Y	D V **N**	**G A** Y A G S **S N** W V	IGLV2-11*01
Heavy chain	**D A** S I S S **N** S **F** Y	**M R** Y S G S T	**T** R W S D G P Y G F D I	IGHV4-39*01

Amino acid sequences of heavy and light chain CDR regions of HuMab-28–009 and their top matching germline genes. Mutated amino acids compared to germline sequence are highlighted in bold.

In protection of mice against Mtb, human IgG2, IgG3 and IgG4 but not IgG1 were protective (Fig. 4b). A study comparing human IgG and murine IgG subclasses against murine Fc receptors demonstrated a comparable binding affinity and pattern, suggesting that human IgG subclass antibodies could have similar FcγR mediated biological activities in mice^41^. In particular, studies have also intimated that human IgG2 antibodies have binding affinity for murine FcγRIII^41^, which is an ortholog of human FcγRIIa^42^, suggesting a possible role for human FcγRIIa in mediating protection. Dimeric murine IgA also demonstrated moderate protection in vivo (Fig. 4e). These findings were significant considering that the Mtb bacterial reduction was from a single dose of antibody and without co-administration of cytokines, as were required in a prior study of Mtb-specific IgA^9^. Incidentally, this study is the first demonstration that LpqH-specific monoclonal antibody can be protective against Mtb.

By contrast, in the human ex vivo MGIA, only human IgG2 was protective (Fig. 4d). A study of LAM-specific IgGs from both asymptomatic and active TB patients also revealed a potential protective role for IgG2^21^. Human IgG2 is associated with other bacterial infections where high IgG2 titres induce enhanced phagocytosis in an FcγRIIa-dependent manner and regulate survival in acute melioidosis^43^. It has also been demonstrated that the human IgG2 subclass elicits a Th17 skewed proinflammatory cytokine response marked by upregulated expression of TNFα, IL1β, IL23 which are dependent on FcγRIIa activation via IRF5 mediated glycolytic changes^44^. Although the mechanism by which IgG2 shows greater protection is not known, the stimulation of Th17 responses, which has been implicated in protection against TB, is potentially intriguing^45,46^.

Our study identifies LpqH as a potential vaccine candidate antigen from an unbiased holistic antibody screen using intact bacteria. A study using Mtb H37Rv membrane vesicles (MV) rich in lipoproteins given subcutaneously, revealed that the immunization generated a strong antibody response to LpqH and also demonstrated increased protective efficacy when Mtb MV boosters were given to BCG-vaccinated mice^47^. A study including LpqH as one of the candidates in a multi-epitope peptide-based vaccine strategy has demonstrated protection against Mtb^48^. However, currently, none of the subunit vaccine strategies in clinical trials include LpqH^49,50^. Our work supports the evaluation of LpqH as a vaccine candidate.

Our study identifies a human monoclonal antibody HuMab-28-009 against an Mtb virulence factor LpqH, from the IgA repertoire of a naturally exposed asymptomatic individual demonstrating moderate protection against Mtb. These findings are suggestive of the role of mucosal antibodies in early host defense, during the initial stages of infection and in preventing progression to active disease. The protective role of human IgG2 and murine IgA, adds significance to antibody therapeutic and prophylactic strategies in TB. Unbiased screens for potentially protective antibodies have promise in identifying potential vaccine candidate antigens for TB, as well as a source for therapeutic antibodies in adjunct treatment of TB^51^.

## Methods

### Ethics statement

The study was approved by the institutional review board of the Beijing Tumor and Thoracic Chest Hospital, China (IRB 2014-2-25) and written informed consent was obtained from donors before sample collection for the antibody screening and whole blood assays performed. Mice were maintained in accordance with the institutional protocols for experiments performed at Beijing Tumour and Thoracic Chest Hospital, China and University of California, San Francisco, USA. Approvals for animal work and standard safety procedures for biosafety level-3 (BSL-3) work were followed according to institutional protocols: Beijing Tumor and Thoracic Chest Hospital, China and University of California, San Francisco, USA.

### Antibody screening and discovery

Memory B cells (CD19+, CD27+ and IgG+ or IgA+) from the donor PBMC sample was isolated and cultured in 384-well plates for 6 days on a layer of irradiated CD40L expressing feeder cells, in a mixture of cytokines such as Interleukins 2, 6, 10 and 15 that induces their differentiation into antibody-secreting plasma cells to propagate antibodies. The culture supernatants from these memory B cell clones were tested for IgG and IgA antibodies binding to whole cell Mtb H37Rv strain, by indirect ELISA (384-well plate format). Six high binding B cell clones were selected from the 384-well plate ELISA screening, for the downstream process. From the selected B cell clones, mRNA was extracted, followed by reverse transcription polymerase chain reaction (RT-PCR) of the variable heavy and light chain genes and their antibody sequences analysed by next-generation sequencing (NGS) to identify unique CDR3 sequences. We identified 12 unique heavy chain and 22 unique light chain sequences. The heavy and light chain sequences identified from each clone were then paired into all possible combinations to identify the original heavy/light chain combination that made up the antibody from each clone (Supplementary Table 1). Fifty-six heavy chain/light chain combinations representing all of the possible original pairings (Supplementary Table 1) were expressed as IgG1 for secondary screening. Small scale transfection of the heavy/light chain plasmids was carried out and harvested five days post transfection. Transfected supernatants of heavy/light chain pairs were subjected to a secondary ELISA screen to identify those that show significant binding to Mtb. Isotype-specific primers for the heavy chain and kappa/lambda specific primers for the light chain were utilized during RT-PCR of the selected high binder clones. From the bioinformatics sequencing analysis, the original isotype and the natural light chain type of the identified antibody HuMab-28-009 was determined (Data availability—Figshare 10.6084/m9.figshare.23549964).

### 384-well plate ELISA

Gamma-irradiated whole cell bacteria Mtb H37Rv (Cat. No. NR-49098) obtained from BEI resources, United States, was resuspended in sterilized Milli Q water and adjusted to OD_600_ of 1.0 for antigen coating of the ELISA plates (Nunc Immuno Maxisorp). Bacterial solution of 20 µL was added, followed by heat fixation at 65 °C for 3 h. Ice cold 70% methanol of 20 µL volume was added and incubated for 2 h at 4 °C. After incubation, the methanol was gently removed and 20 µL of 1X PBS was added to the plates and left overnight at 4 °C. Indirect ELISA was carried out the following day, by blocking with 20 µL of 4% skim milk (Sigma-Aldrich, United States, Cat. No. 70166) in 1X PBS for 4 h, followed by 20 µL of block solution containing 5 µL of B cell culture supernatant for 1 h. This was followed by addition of 20 µL secondary antibody, anti-human IgG-HRP (Thermofisher Scientific, United States, Cat. No. 31413) or anti-human IgA HRP (Thermofisher Scientific, United States, Cat. No. A18781) in 1:5000 added in block solution for 1 h. The plates were read at OD 450 nm after addition of TMB substrate (Thermofisher Scientific, United States, Cat. No. 34029) and stopping the reaction with 1 M concentrated H_2_SO_4_ after 5 min. All washing steps in between were done with 100 µL volume of 1X PBS thrice (Data availability—Figshare 10.6084/m9.figshare.23549964).

### 96-well plate ELISA

Antigens for serological profile testing, HspX (Cat. No. NR-49428), LpqH (Cat. No. NR-50757), CFP10 (Cat. No. NR-49425), ESAT6 (Cat. No. 49424), PstS1 (Cat. No. NR-14859), Ag 85 complex (Cat. No. 14855), Mtb strains: H37Rv (Cat. No. NR-49098), HN878 (Cat. No. NR-49100), CDC1551 (Cat. No. NR-49099), East African Indian 91_0079 (Cat. No. NR-36492) and Indo-Oceanic T17X (Cat. No. NR-36491), were obtained from BEI resources, United States. Mtb antigenic fractions: cell wall (Cat. No. NR-14828), cell membrane (Cat. No. NR-14831), cytosol (Cat. No. NR-14834), protein (Cat. No. NR-14841), Lipoarabinomanan (LAM) (Cat. No. NR-14848) and Lipomannan (LM) (Cat. No. NR-14850) were obtained from BEI resources, United States. Recombinant Mtb protein LpqE (Cat. No. MBS1234581), HBHA (Cat. No. MBS1038591) were purchased from MyBioscources, United States. Antigenic fraction and proteins were constituted in 1X PBS at a concentration of 2 µg/mL, added to the 96-well ELISA plate (Nunc Immuno Maxisorp) at 100 µL/well and incubated at 4 °C overnight. For the whole-cell bacterial ELISA, bacterial solution of OD_600_ of 1.0 of 100 µL was added, followed by heat fixation at 65 °C for 3 h. Subsequently, ice cold 70% methanol of 100 µL volume was added and incubated for 2 h at 4 °C. After incubation, the methanol was gently removed and 100 µL of 1X PBS was added to the plates and left overnight at 4 °C.

Indirect ELISA was carried out the following day, by blocking with 4% skim milk (Sigma-Aldrich, United States, Cat. No. 70166) in 1X PBS for 2 h, followed by primary antibody at respective concentration and secondary antibody, anti-human IgG-HRP (Invitrogen, Cat. No. 31413) in 1:5000 added in block solution. For the serological profiling ELISA, purified Igs of donor samples were diluted in 1:5 ratio in block solution. For the secondary ELISA screening, the transfected supernatants of heavy/light chain pairs were diluted in 1:1 ratio in block solution. The plates were read at OD 450 nm after addition of TMB substrate (Thermo Scientific, Cat. No. 34029) and stopping the reaction with 1 M concentrated H_2_SO_4_ after 5 min. All incubations were for 1 h at room temperature with 100 µL volume and washing steps in between with 300 µL 1X PBS thrice (Data availability—Figshare 10.6084/m9.figshare.23549964, 10.6084/m9.figshare.23550108).

### Antibody engineering and expression

The antibody expression vectors were constructed on the pTT5 vector (Fisher Scientific, United States, Cat. No. 50-148-474) backbone. Separate expression vectors were made for the heavy chain that contain constant regions human IgG1, IgG2, IgG3, IgG4 and murine IgG1, IgA. Separate expression vector was made for light chain containing the lambda constant region. The variable heavy and light chain antibody sequences were cloned into the respective expression vector. The respective heavy and light chain plasmids were transfected into HEK293-6E cells (ATCC, United States) using branched polyethyleneimine (PEI) (Sigma-Aldrich, United States, Cat. No. 408727) as a transfection reagent. The heavy and light chain plasmids of 0.4 μg/mL each and 2.4 μg/mL of PEI (PEI: DNA in 3:1 ratio) were mixed in filter sterilized 150 mM NaCl solution at 1/10th of cell culture volume. This solution was incubated for 5 min and added to the cell culture. This transfected cell culture was maintained in a shaking incubator at 37 °C, 5% CO_2_, 125 rpm. Tryptone-N1 (Organotechnie, France, Cat. No. 19553) at a concentration of 0.5% in total volume of culture was added the following day, to induce protein expression. The cell culture supernatant was harvested after 1 week, to recover the antibody. The antibodies were purified using the 5 mL Protein G HiTrap column (GE Healthcare, United Kingdom, Cat. No. 17-0405-01) for the IgG antibodies and lambda select HiTrap column (GE Healthcare, United States, Cat. No. 17548212) for murine IgA antibody by AKTA pure FPLC system (GE Healthcare, United Kingdom). The peak fraction was collected and buffer exchanged into 1X PBS with an Amicon 100000 MWCO concentrator (GE Vivaspin20 100KDa MWCO Cat. No. 28-9323-63). Antibody expression gel images shown in Fig. 3b, Supplementary Fig 2 are from the same experiment, and they were processed in parallel (Data availability—Figshare 10.6084/m9.figshare.23550117).

### Flow cytometry for B and T cell immunoprofiling

Donor PBMCs was stained with live-dead marker (1:1000; Invitrogen, LIVE/DEAD™ Fixable Near-IR Dead Cell Stain Kit) to gate out the dead cells for 20 min in 4 °C. After washing with FACS buffer, the cells were stained with respective B, T cell marker panel antibodies diluted in FACS buffer (1xPBS, 2% FBS, 0.1% sodium azide) at 4 °C for 30 min. B cell panel: CD19-BV510 (1:40; Biolegend, Cat. No. 363020), CD14-APC-Cy7 (1:50; BD, Cat. No. 557831), CD3-APC-Cy7 (1:125; Biolegend, Cat. No. 317342), CD10-PE-Dazzle594 (1:50; Biolegend, Cat. No. 312228), CD21-APC (1:50; Biolegend, Cat. No. 354906), CD27-BV650 (1:40; Biolegend, Cat. No. 302828), CD38-PE-Cy7 (1:40; Biolegend, Cat. No. 356608), PD-1-BV711 (1:20; Biolegend, Cat. No. 329928), IgD-BUV 737 (1:40; BD, Cat. No. 612798), IgG-PerCP (1:40; BD), IgA-PE (1:40; Miltenyi, Cat. No. 130-114-002). T cell panel: CD14-APC-Cy7 (1:50; BD, Cat. No. 557831), CD19-APC-Cy7 (1:50; BD, Cat. No. 557791), CD3-BUV395 (1:40; BD, Cat. No. 563546), CD4-BUV496 (1:40; BD, Cat. No. 612936), CD8-PE (1:40; BD, Cat. No. 555367), CXCR5-PE-Cy7 (1:100; BD, Cat. No. 624052), CD25-BV421 (1:40; BD, Cat. No. 562442), CD127-FITC (1:20; BD, Cat. No. 557938). The samples were then washed, resuspended in FACS buffer and analysed on BD LSRFortessa flow cytometer (Data availability—Figshare 10.6084/m9.figshare.23549964).

### Flow cytometry for antibody binding to bacteria

Mycobacterial species *M. malmoense*, *M. simiae*, *M. chelonae* and *M. smegmatis* were grown in 7H9 culture media at 37 °C. Bacteria was harvested at OD_600_ of 0.8, washed and resuspended in 1X PBS to OD_600_ of 0.4. It was incubated with antibody HuMab-28-009 at a concentration of 5 µg/mL for 1 h at 4 °C. The bacteria were then washed with FACS buffer (1X PBS, 2% FBS, 0.1% sodium azide) and incubated with Alexa Fluor 647 anti-human IgG (Thermo Scientific Cat. No. A-21445) at 1:200 in FACS buffer for 30 min, followed by washing with FACS buffer to remove the unbound. The bacterial samples were then fixed with 4% formaldehyde solution for 15 min at room temperature and analysed on BD LSRFortessa flow cytometer (Data availability—Figshare 10.6084/m9.figshare.23550108).

### Immunoprecipitation and western blot for antigen identification

The Mtb protein fraction at 200 µg/mL was first pre-absorbed with irrelevant antibody 3CH5 (anti-dengue antibody) and immunoprecipitated with 2 µg/mL of HuMab-28-009 using protein G sepharose beads (GE, United States, Cat. No. 17-0618-02). The immunoprecipitated product (IP) was run on a 12% SDS-PAGE gel in reducing conditions, along with 3 µg of denatured Mtb protein fraction and cell membrane fraction, followed by transfer of the proteins to nitrocellulose membrane (Bio-Rad, United States, Cat. No.162-0115) by immunoblotting. A blocking solution of 5% skim milk (Sigma-Aldrich, United States, Cat. No. 70166) in 1X TBST was prepared, and the blot was blocked for 1 h. The immune blot was then probed with 0.5 µg/mL of HuMab-28-009 in block solution, overnight at 4 °C. The blot was washed and incubated for 1 h with anti-human IgG-HRP (Invitrogen, United States, Cat. No. 31413) in a dilution of 1:18,000 in block solution. The blot was washed and visualized by chemiluminescence method, with substrate Western Bright ECL (Advansta, United States, Cat. No. R03031-D25) and Western Bright peroxide (Advansta, United States, Cat. No. R03025-D25) in a 1:1 ratio for 5 s exposure time. The blots were washed five times with 1X TBST for 5 min each time, after primary and secondary antibody incubations. Blot image shown in Fig. 2d is derived from the same experiment, and they were processed in parallel (Data availability—Figshare 10.6084/m9.figshare.23550108).

### Dot blot analysis

A peptide library of the LpqH protein sequence consisting of 15-mer peptides with 10-mer overlap was created (Genescript Inc). 4 µg of each peptide was spotted on to a nitrocellulose membrane. Once the membrane was dry, it was blocked with 5% skim milk in 1X TBST for 1 h followed by probing with 10 µg/mL of HuMab-28-009 (human IgG1) in block solution at 4 °C overnight. The blot was then washed and probed with anti-human IgG (1:1000) in block solution for 1 h. The blot was finally visualized by chemiluminescence method of detection, with substrate Western Bright ECL (Advansta, United States, Cat. No. R03031-D25) and Western Bright peroxide (Advansta, United States, Cat. No. R03025-D25) in a 1:1 ratio for 15 s exposure time. The blots were washed with 1X TBST five times after primary and secondary antibody incubation. Blot image shown in Supplementary Fig 4 is derived from the same experiment, and they were processed in parallel (Data availability—Figshare 10.6084/m9.figshare.23550108).

### Antibody binding kinetics

Antibody kinetics and affinity was measured using an Attana Cell A200 (Attana AB) at 22 °C. LpqH antigen was immobilized to the LNB-carboxyl sensor chip (Cat. No. 3623-3103) by amine coupling procedure where one chip was coated (channel A) with LpqH antigen and the other chip (channel B) served as reference. Attana LNB-carboxyl sensor chips (A and B channel) were activated with sulfo-NHS/EDC and saturated with LpqH antigen at 3 µg/mL diluted in sodium acetate buffer pH 5.6 (A channel). Ethanolamine was injected to deactivate the chip surface (A and B channel). A pulse of 300 s was used to activate and deactivate the chip. HBST was used as the running buffer. Conditions of injection and regeneration of the chip, with different antibody concentrations, was optimized. After optimal conditions were identified, cFAST automated experimental setup, with different concentrations in triplicates of each antibody was done. Antibody injections were applied as 84 s pulses with dissociation for 400 s at a flow rate of 25 μL/ min for the human and murine IgG antibodies. Regeneration of the chip was done by an 8 s pulse of 50 mM sodium bicarbonate, pH 11 for human IgG1, IgG2, IgG3 and 20 s pulse for human IgG4, murine IgG1 antibodies. Antibody injections were applied as 84 s pulses with dissociation for 300 s at a flow rate of 25 µL/min for murine IgA dimer antibody. Regeneration of the chip was done by 35 s pulse of 50 mM sodium bicarbonate, pH 11 for murine IgA dimer antibody. Curve fitting and subsequent data analysis was performed using TraceDrawer software (Ridgeview Instruments) to determine the association equilibrium (K_a_), disassociation equilibrium (K_d_) and binding affinity (K_D_) (Data availability—Figshare 10.6084/m9.figshare.23550117).

### In vivo murine protection assay

For the in vivo murine protection assay, two doses of 0.1 mg and 0.05 mg of the monoclonal antibody per mouse was injected via intraperitoneal route into BALB/c mice, 5 h before Mtb infection. The HN878 Beijing strain was used for infection via aerosolization. All mice per batch experiment, were infected at the same time using a Glas-col inhalation exposure system, with an infecting dose of 20–30 CFU per mouse (Day 0). The mice were euthanized after fourteen days, and lung homogenates plated in 7H11 agar plates supplemented with 10% OADC. The bacterial burden was determined after three weeks of incubation at 37 °C. For the in vivo assay with purified total Igs, 20 mg dose was used per mouse and Beijing strain 10165 was used, with an infecting dose of 100–200 CFU per mouse (24 h post infection) (Data availability—Figshare 10.6084/m9.figshare.23550129, 10.6084/m9.figshare.23549964).

### Ex vivo whole blood mycobacterial growth inhibition assay (MGIA)

Healthy donor whole blood from multiple donors was diluted with RPMI-1640 at 1:1 ratio. This was then spiked with a locally isolated Beijing strain 10165 at 10^5^ CFU and antibody at a concentration of 5 µg/mL, in triplicate. The samples were incubated at 37 °C in a shaking incubator at 20 rpm for 96 h, followed by RBC lysis. The samples were then centrifuged to remove the supernatant and the WBC cell pellet was resuspended in 1 mL PBS. The cell pellet was serially diluted and plated in 7H10 agar plates supplemented with 10% OADC, followed by CFU enumeration after three weeks of incubation at 37 °C. For the assay with purified total Igs, 50 µg/mL concentration was used, with an incubation period of 120 h (Data availability—Figshare 10.6084/m9.figshare.23550129, 10.6084/m9.figshare.23549964).

### Data analysis

For the statistical analysis of ELISAs, ex vivo whole blood MGIA and in vivo murine protection assay experiments, means of different groups were compared to control group using ordinary one-way ANOVA, with Dunnett’s multiple comparisons test, using GraphPad Prism version 9 and *P* < 0.05 were considered statistically significant. Two-tailed student T-tests were used for in vivo murine and ex vivo whole blood MGIA with donor Igs.

### Reporting summary

Further information on research design is available in the Nature Research Reporting Summary linked to this article.

### Supplementary information

Supplementary Material

REPORTING SUMMARY

## Data Availability

All data supporting the findings of this study are available within the paper, supplementary Information, and general repository Figshare 10.6084/m9.figshare.23549964, 10.6084/m9.figshare.23550108, 10.6084/m9.figshare.23550117, 10.6084/m9.figshare.23550129).
