# Author Correction: An anti-LpqH human monoclonal antibody from an asymptomatic individual mediates protection against *Mycobacterium tuberculosis*

**DOI:** 10.1038/s41541-023-00735-6

**Published:** 2023-09-25

**Authors:** Shivankari Krishnananthasivam, Hao Li, Rania Bouzeyen, Bhuvaneshwari Shunmuganathan, Kiren Purushotorman, Xinlei Liao, Fengjiao Du, Claudia Guldager Kring Friis, Felicity Crawshay-Williams, Low Heng Boon, Qian Xinlei, Conrad En Zuo Chan, Radoslaw Sobota, Mary Kozma, Valeria Barcelli, Guirong Wang, Hairong Huang, Andreas Floto, Pablo Bifani, Babak Javid, Paul A. MacAry

**Affiliations:** 1 Department of Microbiology and Immunology, Yong Loo Lin School of Medicine, National University of Singapore, Singapore, Singapore; 2 College of Veterinary Medicine, China Agricultural University, Beijing, China; 3 Center for Infectious Disease Research, School of Medicine, Tsinghua University, Beijing, China; 4 Division of Experimental Medicine, University of California, San Francisco, USA; 5 Life Sciences Institute, National University of Singapore, Singapore, Singapore; 6 National Clinical Laboratory on Tuberculosis, Beijing Tuberculosis and Thoracic Tumor Institute, Beijing Chest Hospital, Capital Medical University, Beijing, P.R. China; 7 Molecular Immunity Unit, University of Cambridge, Department of Medicine, MRC Laboratory of Molecular Biology, Cambridge, UK; 8 National Centre for Infectious Diseases, Tan Tock Seng Hospital, Singapore, Singapore; 9 Defence Medical and Environmental Research Institute, DSO National Laboratories, Singapore, Singapore; 10 Institute of Molecular and Cell Biology (IMCB), Agency for Science, Technology and Research (A*STAR), Singapore, Singapore

**Keywords:** Biomarkers, Tuberculosis

Correction to: *npj Vaccines* 10.1038/s41541-023-00710-1, published online 25 August 2023

In this article, the author name Valeria Barcelli was incorrectly written as Valeria Barcelli Jo. The original article has been corrected.

