# Supplementary material for: An anti-LpqH human monoclonal antibody from an asymptomatic individual mediates protection against Mycobacterium tuberculosis
Source: NPJ Vaccines. 2023 Aug 25;8:127. doi: 10.1038/s41541-023-00710-1 (PMC10457302; doi:10.1038/s41541-023-00710-1)
Supplement: Supplementary file 1 — Supplementary Material [file 41541_2023_710_MOESM1_ESM.pdf]

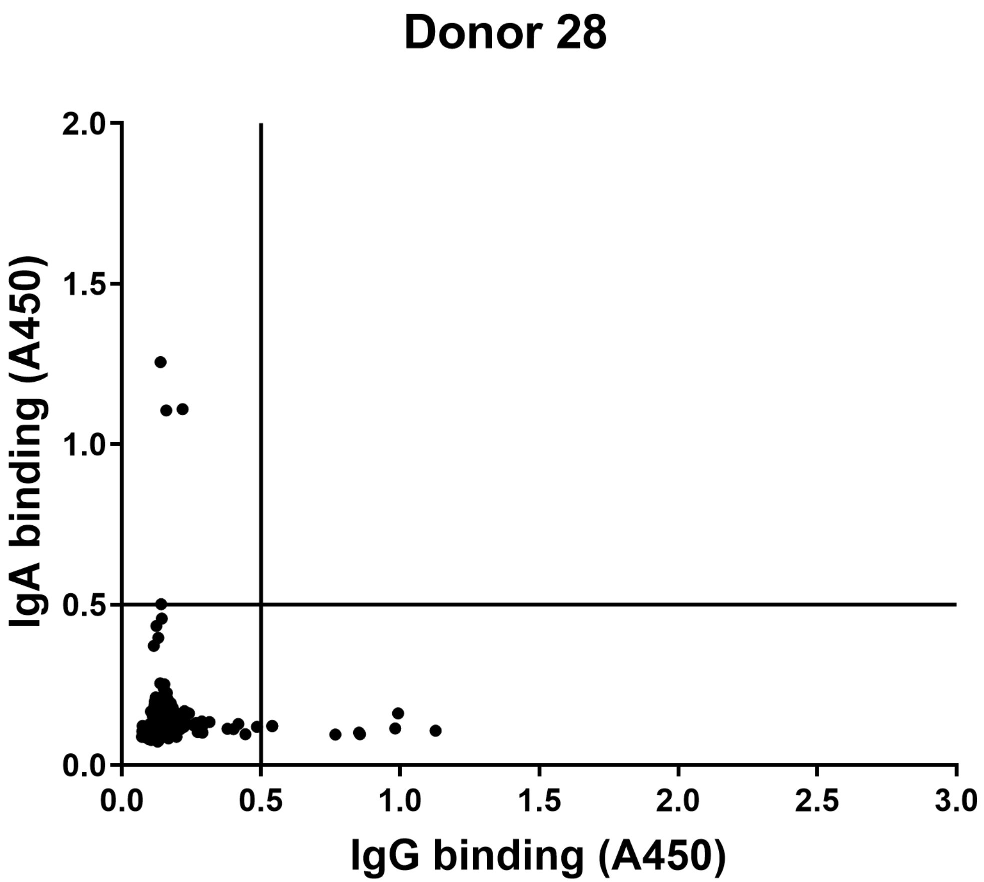


**Supplementary Figure 1: Antibody screening.** IgG and IgA screening of activated memory B cell culture supernatant samples of donor 28 against gamma-irradiated whole cell Mtb by indirect ELISA.

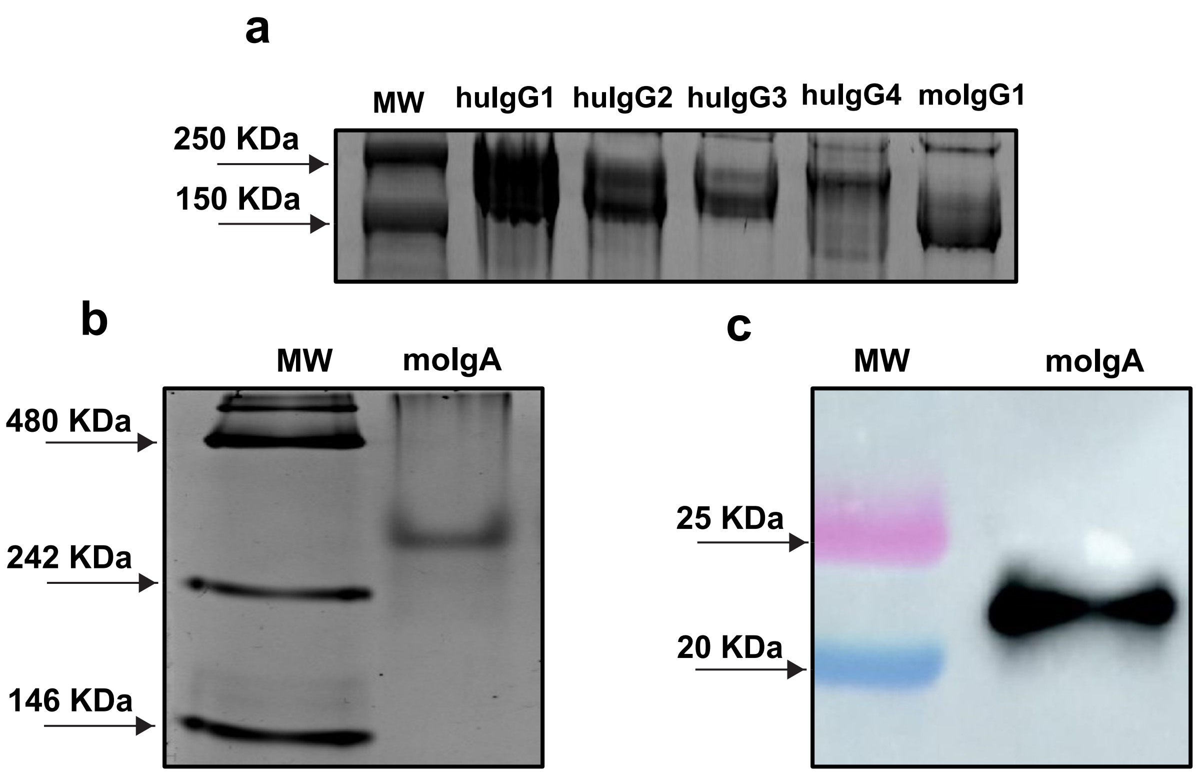


**Supplementary Figure 2: Antibody expression of human, murine IgG subclass and murine IgA.** (a) Non-reducing SDS-PAGE gels showing fully assembled IgG antibodies around 150 KDa confirming antibody expression. (b) Native gel confirms assembly of murine IgA dimer above 300 KDa. (c) Western blot shows expression of the murine J chain above 15 KDa.

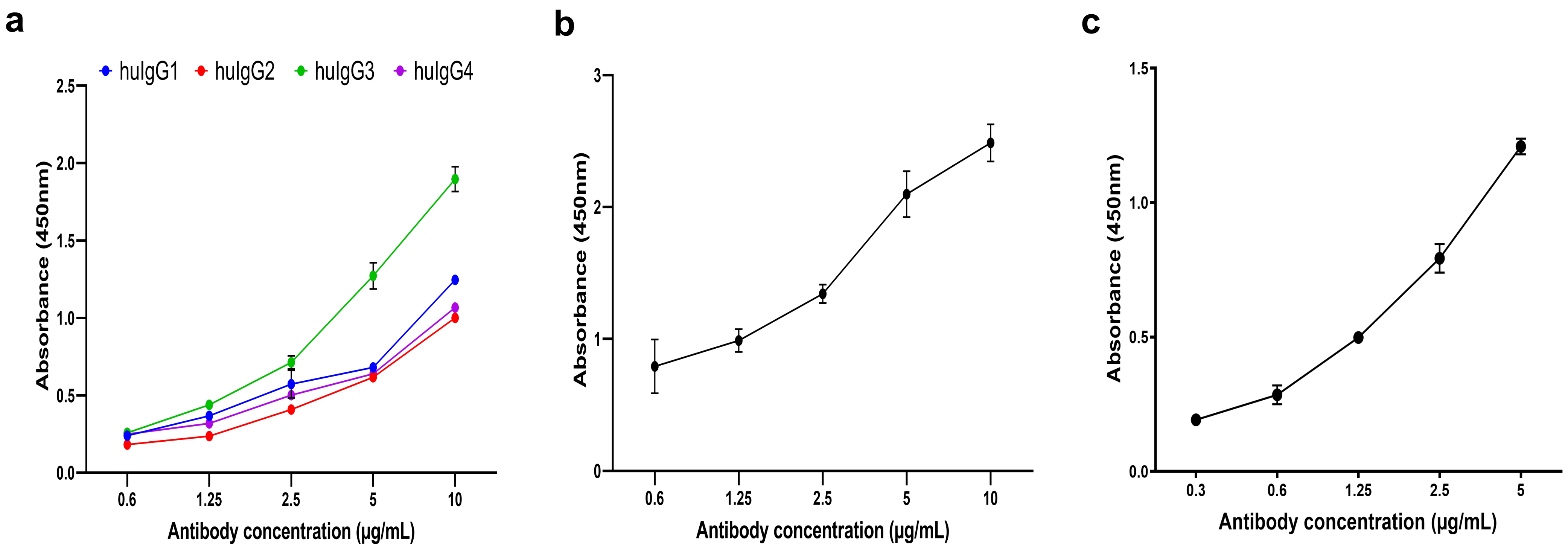


**Supplementary Figure 3: Binding activity of human, murine IgG subclass and murine IgA.** Indirect ELISA showing binding activity of (a) human IgG subclass (b) murine IgG1 (c) murine IgA antibodies against LpqH.

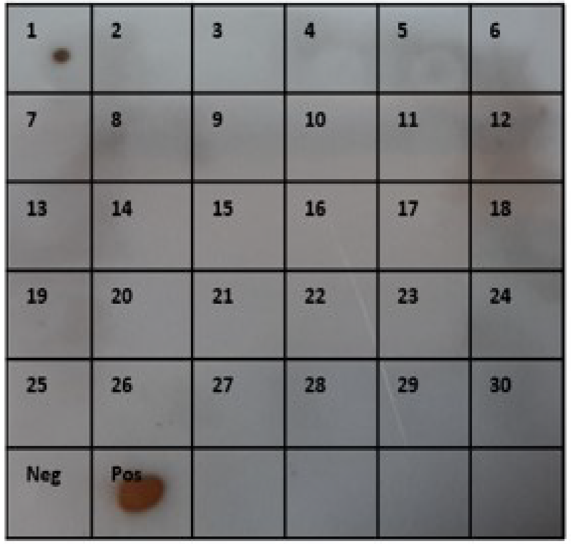


**Supplementary Figure 4: Overlapping peptide library scanning.** Dot blot analysis of the LpqH peptide library, consisting of 15 mers with a 10 mer overlap, shows binding of HuMab-28-009 to peptide 1. Recombinant LpqH protein was used as the positive control and DMSO was used as the negative control.

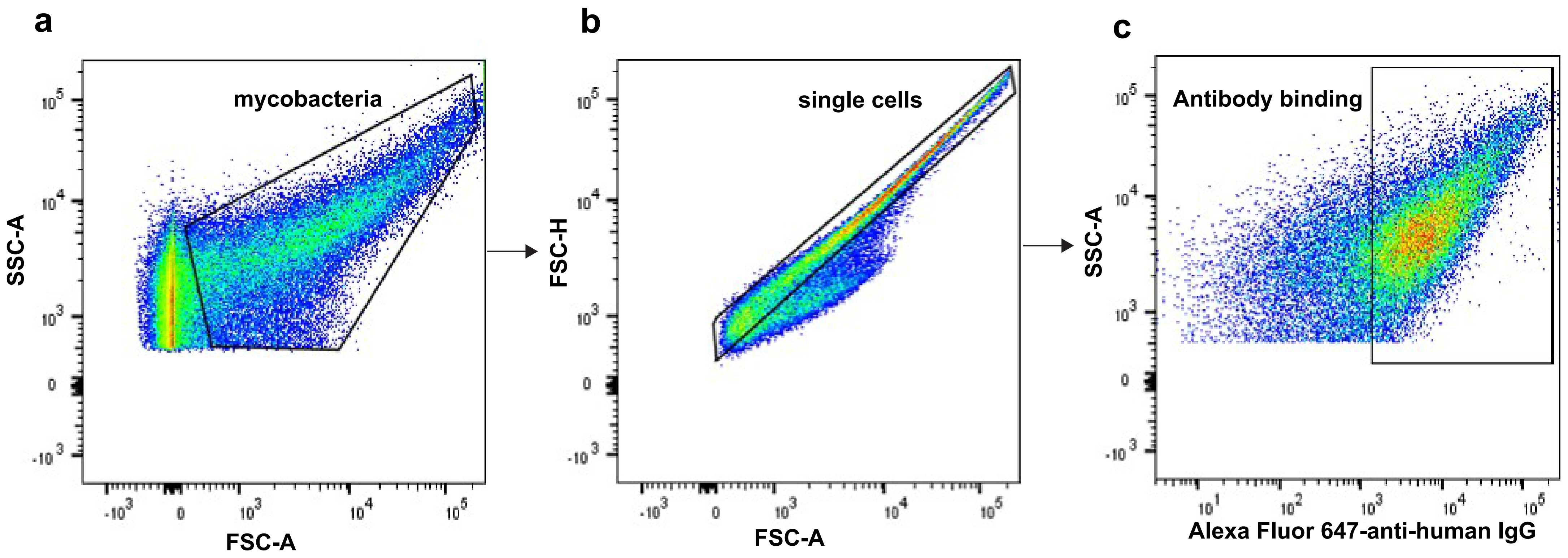


**Supplementary Figure 5: Gating strategy for assessment of binding activity of HuMab-28-009 to mycobacteria.** (a) Mycobacteria are gated based on FSC-A and SSC-A parameters, followed by gating for (b) single cells based on FSC-A and FSC-H parameters. The binding activity of HuMab-28-009 and isotype control is then assessed by gating based on (c) SSC-A and Alexa Fluor 647-anti-human IgG.

**Supplementary Table 1: Heavy-light chain pairings of the identified antibody sequences from six high binder B cell clones.** The six unique high-binding B cell clones identified are P2K, P1C, P1F, P1I, P2D, and P2L. HC refers to a unique heavy chain variable sequence and LC refers to a unique light chain variable sequence.

| P2K | HC1 | HC2 | | P1C | HC1 |
|---|---|---|---|---|---|
| LC1 | 1 | 2 | | LC1 | 5 |
| LC2 | 3 | 4 | | | |

| P1F | HC1 | HC2 | HC3 |
|---|---|---|---|
| LC1 | 6 | 7 | 8 |
| LC2 | 9 | 10 | 11 |
| LC3 | 12 | 13 | 14 |
| LC4 | 15 | 16 | 17 |
| LC5 | 18 | 19 | 20 |
| LC6 | 21 | 22 | 23 |
| LC7 | 24 | 25 | 26 |
| LC8 | 27 | 28 | 29 |

| P1I | HC1 |
|---|---|
| LC1 | 30 |
| LC2 | 31 |

| P2D | HC1 | HC2 | HC3 |
|---|---|---|---|
| LC1 | 32 | 33 | 34 |
| LC2 | 35 | 36 | 37 |
| LC3 | 38 | 39 | 40 |
| LC4 | 41 | 42 | 43 |
| LC5 | 44 | 45 | 46 |
| LC6 | 47 | 48 | 49 |
| LC7 | 50 | 51 | 52 |

| P2L | HC1 | HC2 |
|---|---|---|
| LC1 | 53 | 54 |
| LC2 | 55 | 56 |

**Supplementary Table 2: Antibody epitope mapping by peptide library scanning.** Amino acid sequences of relevant peptides, with N terminal VKRGL sequence of peptide 1 showing binding to HuMab-28-009.

| Peptide No: | Amino acid sequence | Amino acid No: |
|---|---|---|
| 1 | VKRGLTVAVAGAAIL | 1-15 |
| 2 | TVAVAGAAILVAGLS | 6-20 |

**Supplementary Table 3: Accession numbers of the 630 Mtb complex isolates used in phylogenetic analysis.**

| Accession | Sample | Lineage |
|---|---|---|
| ERR718222 | ERX662144 | L1 |
| ERR751673 | ERX695355 | L1 |
| SRR5065256 | SRX2385623 | L1 |
| SRR5065402 | SRX2385768 | L1 |
| SRR5065509 | SRX2385875 | L1 |
| SRR5067498 | SRX2387796 | L1 |
| SRR5067667 | SRX2387965 | L1 |
| SRR5073812 | SRX2392902 | L1 |
| SRR5073977 | SRX2393067 | L1 |
| SRR5074133 | SRX2393222 | L1 |
| SRR6153022 | SRX3264814 | L1 |
| ERR718341 | ERX662263 | L1 |
| ERR767967 | ERX711240 | L1 |
| ERR768007 | ERX711280 | L1 |
| ERR550626 | ERX509695 | L1 |
| ERR768021 | ERX711294 | L1 |
| SRR5074057 | SRX2393148 | L1 |
| ERR718375 | ERX662297 | L1 |
| ERR718547 | ERX662469 | L1 |
| ERR751900 | ERX695582 | L1 |
| ERR752066 | ERX695748 | L1 |
| ERR767980 | ERX711253 | L1 |
| ERR751749 | ERX695431 | L1 |
| ERR752198 | ERX695880 | L1 |
| ERR768006 | ERX711279 | L1 |
| ERR718371 | ERX662293 | L1 |
| ERR752067 | ERX695749 | L1 |
| ERR752244 | ERX695926 | L1 |
| ERR718220 | ERX662142 | L1 |
| ERR718227 | ERX662149 | L1 |
| ERR751780 | ERX695462 | L1 |
| ERR046856 | ERX023918 | L1 |
| ERR1023339 | ERX1102211 | L1 |
| ERR164002 | ERX139879 | L1 |

| | | |
|---|---|---|
| ERR228169 | ERX202828 | L1 |
| ERR230098 | ERX204649 | L1 |
| ERR234235 | ERX208740 | L1 |
| ERR245698 | ERX220232 | L1 |
| ERR387003 | ERX359233 | L1 |
| SRR5818570 | SRX2996873 | L1 |
| SRR6046489 | SRX3193417 | L1 |
| ERR037521 | ERX1973347 | L1 |
| ERR181862 | ERX157972 | L1 |
| ERR212134 | ERX186799 | L1 |
| ERR245656 | ERX220190 | L1 |
| ERR245695 | ERX220229 | L1 |
| ERR245721 | ERX220255 | L1 |
| ERR245782 | ERX220316 | L1 |
| ERR294219 | ERX267552 | L1 |
| ERR779657 | ERX770084 | L1 |
| ERR1023317 | ERX1102189 | L1 |
| ERR1063834 | ERX1143984 | L1 |
| ERR117679 | ERX093953 | L1 |
| ERR123943 | ERX100125 | L1 |
| ERR230065 | ERX204616 | L1 |
| ERR294179 | ERX267512 | L1 |
| ERR386815 | ERX359045 | L1 |
| ERR718329 | ERX662251 | L1 |
| SRR2100170 | SRX1094864 | L1 |
| SRR2100582 | SRX1095276 | L1 |
| ERR117706 | ERX093980 | L1 |
| ERR234266 | ERX208771 | L1 |
| ERR270665 | ERX244892 | L1 |
| ERR278515 | ERX251848 | L1 |
| ERR386874 | ERX359104 | L1 |
| ERR386888 | ERX359118 | L1 |
| ERR403407 | ERX369768 | L1 |
| SRR5007184 | SRX2338833 | L1 |
| SRR6152641 | SRX3264759 | L1 |
| SRR6153146 | SRX3265191 | L1 |
| ERR718348 | ERX662270 | L1 |
| ERR718394 | ERX662316 | L1 |
| ERR751938 | ERX695620 | L1 |
| ERR718306 | ERX662228 | L1 |
| ERR718326 | ERX662248 | L1 |
| ERR718438 | ERX662360 | L1 |
| ERR718534 | ERX662456 | L1 |
| ERR751962 | ERX695644 | L1 |
| ERR752059 | ERX695741 | L1 |
| ERR752263 | ERX695945 | L1 |

| | | |
|---|---|---|
| SRR5067610 | SRX2387908 | L1 |
| ERR038258 | ERX015454 | L1 |
| ERR046945 | ERX024007 | L1 |
| ERR1023379 | ERX1102251 | L1 |
| ERR114452 | ERX091887 | L1 |
| ERR117667 | ERX093941 | L1 |
| ERR123906 | ERX100088 | L1 |
| ERR228185 | ERX202844 | L1 |
| ERR278584 | ERX251917 | L1 |
| ERR387007 | ERX359237 | L1 |
| ERR473297 | ERX439128 | L1 |
| ERR551739 | ERX510808 | L1 |
| ERR234216 | ERX208721 | L2 |
| ERR234252 | ERX208757 | L2 |
| SRR5065411 | SRX2385777 | L2 |
| SRR5065418 | SRX2385784 | L2 |
| SRR5065560 | SRX2385924 | L2 |
| SRR5067475 | SRX2387773 | L2 |
| SRR5067554 | SRX2387852 | L2 |
| SRR5073499 | SRX2392589 | L2 |
| SRR5073637 | SRX2392727 | L2 |
| SRR5074093 | SRX2393183 | L2 |
| SRR5074182 | SRX2393271 | L2 |
| SRR6152905 | SRX3264931 | L2 |
| ERR1023510 | ERX1102382 | L2 |
| ERR117687 | ERX093961 | L2 |
| ERR1213851 | ERX1286064 | L2 |
| ERR1213859 | ERX1286072 | L2 |
| ERR1873490 | ERX1934015 | L2 |
| ERR502885 | ERX468242 | L2 |
| ERR551086 | ERX510155 | L2 |
| ERR552157 | ERX511226 | L2 |
| SRR4423153 | SRX2245337 | L2 |
| SRR5065419 | SRX2385785 | L2 |
| SRR6480432 | SRX3570350 | L2 |
| SRR671855 | SRX225169 | L2 |
| ERR718352 | ERX662274 | L2 |
| ERR751850 | ERX695532 | L2 |
| ERR751959 | ERX695641 | L2 |
| ERR767969 | ERX711242 | L2 |
| ERR1035103 | ERX1113901 | L2 |
| SRR5067271 | SRX2387569 | L2 |
| SRR5073736 | SRX2392826 | L2 |
| ERR502887 | ERX468244 | L2 |
| ERR502919 | ERX468276 | L2 |
| ERR551991 | ERX511060 | L2 |

| | | |
|---|---|---|
| ERR751773 | ERX695455 | L2 |
| ERR552191 | ERX511260 | L2 |
| SRR5073853 | SRX2392943 | L2 |
| SRR671788 | SRX225102 | L2 |
| SRR5153924 | SRX2472120 | L2 |
| ERR117458 | ERX093732 | L2 |
| ERR137195 | ERX113213 | L2 |
| ERR133837 | ERX109855 | L2 |
| ERR144584 | ERX120512 | L2 |
| ERR1023401 | ERX1102273 | L2 |
| ERR1023459 | ERX1102331 | L2 |
| ERR1023512 | ERX1102384 | L2 |
| ERR1633829 | ERX1704162 | L2 |
| ERR234121 | ERX208626 | L2 |
| ERR234140 | ERX208645 | L2 |
| ERR279521 | ERX252854 | L2 |
| ERR551693 | ERX510762 | L2 |
| SRR2024943 | SRX1027336 | L2 |
| SRR5065381 | SRX2385748 | L2 |
| SRR5065665 | SRX2386029 | L2 |
| SRR6152947 | SRX3264889 | L2 |
| ERR234213 | ERX208718 | L2 |
| ERR751652 | ERX695334 | L2 |
| SRR5067541 | SRX2387839 | L2 |
| SRR5074154 | SRX2393243 | L2 |
| SRR6045828 | SRX3193058 | L2 |
| SRR6480459 | SRX3570323 | L2 |
| ERR036210 | ERX1995646 | L3 |
| ERR038300 | ERX015496 | L3 |
| ERR046965 | ERX024027 | L3 |
| ERR046968 | ERX024030 | L3 |
| ERR072077 | ERX049843 | L3 |
| ERR1023324 | ERX1102196 | L3 |
| ERR1023361 | ERX1102233 | L3 |
| ERR114443 | ERX091878 | L3 |
| ERR163966 | ERX139843 | L3 |
| ERR212146 | ERX186811 | L3 |
| ERR046810 | ERX023872 | L3 |
| ERR117671 | ERX093945 | L3 |
| ERR270781 | ERX245008 | L3 |
| ERR278560 | ERX251893 | L3 |
| ERR278588 | ERX251921 | L3 |
| ERR330717 | ERX303646 | L3 |
| ERR330743 | ERX303672 | L3 |
| ERR351898 | ERX324681 | L3 |
| ERR386830 | ERX359060 | L3 |

| | | |
|---|---|---|
| ERR403342 | ERX369703 | L3 |
| ERR433208 | ERX399483 | L3 |
| ERR072084 | ERX049850 | L3 |
| ERR114429 | ERX091864 | L3 |
| ERR114494 | ERX091929 | L3 |
| ERR228141 | ERX202800 | L3 |
| ERR266548 | ERX241044 | L3 |
| ERR266574 | ERX241070 | L3 |
| ERR278586 | ERX251919 | L3 |
| ERR351903 | ERX324686 | L3 |
| ERR403354 | ERX369715 | L3 |
| ERR403380 | ERX369741 | L3 |
| ERR046939 | ERX024001 | L3 |
| ERR072072 | ERX049838 | L3 |
| ERR072076 | ERX049842 | L3 |
| ERR072078 | ERX049844 | L3 |
| ERR072081 | ERX049847 | L3 |
| ERR072097 | ERX049863 | L3 |
| ERR114442 | ERX091877 | L3 |
| ERR270633 | ERX244860 | L3 |
| ERR270729 | ERX244956 | L3 |
| ERR123944 | ERX100126 | L4 |
| ERR133833 | ERX109851 | L4 |
| ERR133874 | ERX109892 | L4 |
| ERR2027300 | ERX2086452 | L4 |
| ERR234198 | ERX208703 | L4 |
| ERR234201 | ERX208706 | L4 |
| ERR234627 | ERX209132 | L4 |
| ERR400495 | ERX366856 | L4 |
| ERR553173 | ERX512242 | L4 |
| ERR751420 | ERX695102 | L4 |
| ERR775384 | ERX718263 | L4 |
| ERR038277 | ERX015473 | L4 |
| ERR038278 | ERX015474 | L4 |
| ERR1023385 | ERX1102257 | L4 |
| ERR751380 | ERX695062 | L4 |
| ERR751451 | ERX695133 | L4 |
| ERR751520 | ERX695202 | L4 |
| ERR751534 | ERX695216 | L4 |
| ERR775322 | ERX718201 | L4 |
| ERR775377 | ERX718256 | L4 |
| ERR779899 | ERX770326 | L4 |
| SRR6046351 | SRX3193555 | L4 |
| ERR039327 | ERX016523 | L4 |
| ERR046741 | ERX023803 | L4 |
| ERR046842 | ERX023904 | L4 |

| | | |
|---|---|---|
| ERR046906 | ERX023968 | L4 |
| ERR046924 | ERX023986 | L4 |
| ERR046962 | ERX024024 | L4 |
| ERR072034 | ERX049800 | L4 |
| ERR1213950 | ERX1286163 | L4 |
| ERR386905 | ERX359135 | L4 |
| SRR2101042 | SRX1095736 | L4 |
| SRR2101227 | SRX1095921 | L4 |
| ERR1023504 | ERX1102376 | L4 |
| ERR046792 | ERX023854 | L4 |
| ERR046793 | ERX023855 | L4 |
| ERR046985 | ERX024047 | L4 |
| ERR1034646 | ERX1113444 | L4 |
| ERR1633802 | ERX1704135 | L4 |
| ERR228254 | ERX202913 | L4 |
| ERR228258 | ERX202917 | L4 |
| ERR386907 | ERX359137 | L4 |
| ERR386969 | ERX359199 | L4 |
| ERR550864 | ERX509933 | L4 |
| ERR553185 | ERX512254 | L4 |
| SRR2101445 | SRX1096139 | L4 |
| ERR1023342 | ERX1102214 | L4 |
| ERR1023440 | ERX1102312 | L4 |
| ERR1023511 | ERX1102383 | L4 |
| ERR234558 | ERX209063 | L4 |
| ERR270684 | ERX244911 | L4 |
| ERR550657 | ERX509726 | L4 |
| ERR552311 | ERX511380 | L4 |
| ERR773788 | ERX716667 | L4 |
| SRR4034343 | SRX2025642 | L4 |
| SRR4035670 | SRX2026827 | L4 |
| SRR4035734 | SRX2026891 | L4 |
| ERR234259 | ERX208764 | L4 |
| ERR551799 | ERX510868 | L4 |
| ERR751383 | ERX695065 | L4 |
| ERR751558 | ERX695240 | L4 |
| ERR760737 | ERX705498 | L4 |
| ERR760740 | ERX705501 | L4 |
| ERR775352 | ERX718231 | L4 |
| SRR5153918 | SRX2472114 | L4 |
| SRR6044829 | SRX3192017 | L4 |
| SRR6045750 | SRX3192626 | L4 |
| SRR6152658 | SRX3264742 | L4 |
| ERR067661 | ERX045524 | L4 |
| ERR067700 | ERX045563 | L4 |
| ERR067757 | ERX045620 | L4 |

| | | |
|---|---|---|
| ERR108472 | ERX085906 | L4 |
| ERR133812 | ERX109830 | L4 |
| ERR133834 | ERX109852 | L4 |
| ERR133911 | ERX109929 | L4 |
| ERR228058 | ERX202717 | L4 |
| SRR3743479 | SRX1897621 | L4 |
| SRR5153854 | SRX2472016 | L4 |
| SRR6152867 | SRX3264969 | L4 |
| ERR046922 | ERX023984 | L4 |
| ERR072049 | ERX049815 | L4 |
| ERR072053 | ERX049819 | L4 |
| ERR228126 | ERX202785 | L4 |
| ERR386950 | ERX359180 | L4 |
| SRR2100799 | SRX1095493 | L4 |
| SRR2101152 | SRX1095846 | L4 |
| SRR5067281 | SRX2387579 | L4 |
| SRR5067435 | SRX2387733 | L4 |
| SRR5073960 | SRX2393050 | L4 |
| ERR028615 | ERX1996942 | L4 |
| ERR028616 | ERX1996943 | L4 |
| ERR028617 | ERX1996944 | L4 |
| ERR257906 | ERX232495 | L4 |
| ERR551034 | ERX510103 | L4 |
| ERR551553 | ERX510622 | L4 |
| ERR067737 | ERX045600 | L4 |
| ERR067767 | ERX045630 | L4 |
| ERR1035182 | ERX1113980 | L4 |
| ERR1213892 | ERX1286105 | L4 |
| ERR144547 | ERX120475 | L4 |
| ERR228036 | ERX202695 | L4 |
| ERR278570 | ERX251903 | L4 |
| ERR751538 | ERX695220 | L4 |
| ERR1193691 | ERX1267004 | L4 |
| ERR230047 | ERX204598 | L4 |
| ERR386977 | ERX359207 | L4 |
| ERR550740 | ERX509809 | L4 |
| ERR551027 | ERX510096 | L4 |
| ERR551474 | ERX510543 | L4 |
| ERR551729 | ERX510798 | L4 |
| ERR551792 | ERX510861 | L4 |
| ERR551875 | ERX510944 | L4 |
| ERR552598 | ERX511667 | L4 |
| ERR553015 | ERX512084 | L4 |
| ERR553018 | ERX512087 | L4 |
| ERR1035344 | ERX1114142 | L4 |
| ERR1213838 | ERX1286051 | L4 |

| | | |
|---|---|---|
| ERR495085 | ERX460501 | L4 |
| ERR751524 | ERX695206 | L4 |
| ERR779867 | ERX770294 | L4 |
| SRR3085354 | SRX1514034 | L4 |
| SRR4034455 | SRX2025754 | L4 |
| SRR4034471 | SRX2025770 | L4 |
| SRR4034489 | SRX2025788 | L4 |
| SRR4034508 | SRX2025807 | L4 |
| SRR4034553 | SRX2025852 | L4 |
| SRR6045304 | SRX3192562 | L4 |
| ERR1023479 | ERX1102351 | L4 |
| ERR1023493 | ERX1102365 | L4 |
| ERR1034992 | ERX1113790 | L4 |
| ERR1034996 | ERX1113794 | L4 |
| ERR279579 | ERX252912 | L4 |
| ERR495036 | ERX460452 | L4 |
| ERR553350 | ERX512419 | L4 |
| ERR966620 | ERX1043598 | L4 |
| SRR2101517 | SRX1096211 | L4 |
| SRR4033424 | SRX2024755 | L4 |
| SRR4037621 | SRX2028743 | L4 |
| ERR108512 | ERX085946 | L4 |
| ERR1193903 | ERX1267216 | L4 |
| ERR133851 | ERX109869 | L4 |
| ERR227990 | ERX202649 | L4 |
| ERR551334 | ERX510403 | L4 |
| ERR552029 | ERX511098 | L4 |
| ERR553210 | ERX512279 | L4 |
| ERR751409 | ERX695091 | L4 |
| ERR751566 | ERX695248 | L4 |
| ERR779855 | ERX770282 | L4 |
| SRR4034580 | SRX2025879 | L4 |
| SRR6045870 | SRX3193016 | L4 |
| ERR228216 | ERX202875 | L4 |
| ERR245745 | ERX220279 | L4 |
| ERR278580 | ERX251913 | L4 |
| ERR386939 | ERX359169 | L4 |
| ERR067693 | ERX045556 | L4 |
| ERR1034881 | ERX1113679 | L4 |
| ERR117450 | ERX093724 | L4 |
| ERR275190 | ERX249512 | L4 |
| ERR275231 | ERX249553 | L4 |
| ERR323086 | ERX296231 | L4 |
| ERR553176 | ERX512245 | L4 |
| ERR751499 | ERX695181 | L4 |
| ERR775329 | ERX718208 | L4 |

| | | |
|---|---|---|
| ERR779874 | ERX770301 | L4 |
| SRR2100531 | SRX1095225 | L4 |
| SRR671775 | SRX225089 | L4 |
| ERR1034847 | ERX1113645 | L4 |
| ERR1035179 | ERX1113977 | L4 |
| ERR1035220 | ERX1114018 | L4 |
| ERR1193835 | ERX1267148 | L4 |
| ERR1664630 | ERX1734941 | L4 |
| ERR1664634 | ERX1734945 | L4 |
| ERR275185 | ERX249507 | L4 |
| ERR275207 | ERX249529 | L4 |
| ERR551880 | ERX510949 | L4 |
| ERR552230 | ERX511299 | L4 |
| ERR553213 | ERX512282 | L4 |
| ERR775350 | ERX718229 | L4 |
| ERR037467 | ERX1973351 | L4 |
| ERR161115 | ERX136965 | L4 |
| ERR163956 | ERX139833 | L4 |
| ERR163998 | ERX139875 | L4 |
| ERR164020 | ERX139897 | L4 |
| ERR176505 | ERX152651 | L4 |
| ERR176555 | ERX152701 | L4 |
| ERR181675 | ERX157785 | L4 |
| ERR736851 | ERX680542 | L4 |
| SRR4035571 | SRX2026728 | L4 |
| ERR1034693 | ERX1113491 | L4 |
| ERR270618 | ERX244845 | L4 |
| ERR351925 | ERX324708 | L4 |
| ERR403402 | ERX369763 | L4 |
| SRR2100055 | SRX1094749 | L4 |
| ERR270680 | ERX244907 | L4 |
| ERR270699 | ERX244926 | L4 |
| SRR4033630 | SRX2024961 | L4 |
| ERR072038 | ERX049804 | L4 |
| ERR228247 | ERX202906 | L4 |
| ERR551609 | ERX510678 | L4 |
| ERR552193 | ERX511262 | L4 |
| ERR553282 | ERX512351 | L4 |
| ERR966621 | ERX1043599 | L4 |
| SRR2502806 | SRX1281290 | L4 |
| SRR4033661 | SRX2024984 | L4 |
| SRR4033682 | SRX2025005 | L4 |
| SRR4034412 | SRX2025711 | L4 |
| SRR4035599 | SRX2026756 | L4 |
| ERR028608 | ERX1996945 | L4 |
| ERR036229 | ERX1995627 | L4 |

| | | |
|---|---|---|
| ERR037541 | ERX1973334 | L4 |
| ERR038282 | ERX015478 | L4 |
| ERR176573 | ERX152719 | L4 |
| ERR270788 | ERX245015 | L4 |
| ERR386984 | ERX359214 | L4 |
| SRR6480597 | SRX3570185 | L4 |
| ERR270624 | ERX244851 | L4 |
| ERR718219 | ERX662141 | L4 |
| ERR751756 | ERX695438 | L4 |
| ERR751978 | ERX695660 | L4 |
| ERR775320 | ERX718199 | L4 |
| SRR1573691 | SRX699682 | L4 |
| SRR5067225 | SRX2387523 | L4 |
| SRR5067575 | SRX2387873 | L4 |
| SRR6153142 | SRX3265195 | L4 |
| SRR671740 | SRX225053 | L4 |
| SRR671861 | SRX225175 | L4 |
| ERR1023362 | ERX1102234 | L4 |
| ERR228143 | ERX202802 | L4 |
| ERR718480 | ERX662402 | L4 |
| ERR752241 | ERX695923 | L4 |
| SRR2100033 | SRX1094727 | L4 |
| SRR6045133 | SRX3192223 | L4 |
| SRR6153240 | SRX3265097 | L4 |
| ERR1023374 | ERX1102246 | L4 |
| ERR1035324 | ERX1114122 | L4 |
| ERR1199119 | ERX1271377 | L4 |
| ERR228111 | ERX202770 | L4 |
| ERR266522 | ERX241018 | L4 |
| ERR266552 | ERX241048 | L4 |
| ERR403338 | ERX369699 | L4 |
| ERR403357 | ERX369718 | L4 |
| ERR403378 | ERX369739 | L4 |
| SRR5818609 | SRX2996834 | L4 |
| ERR117670 | ERX093944 | L4 |
| ERR1199118 | ERX1271376 | L4 |
| ERR234171 | ERX208676 | L4 |
| ERR1023383 | ERX1102255 | L4 |
| ERR1199138 | ERX1271396 | L4 |
| ERR230054 | ERX204605 | L4 |
| ERR234172 | ERX208677 | L4 |
| ERR234173 | ERX208678 | L4 |
| ERR266519 | ERX241015 | L4 |
| ERR330685 | ERX303614 | L4 |
| ERR386814 | ERX359044 | L4 |
| ERR403325 | ERX369686 | L4 |

| | | |
|---|---|---|
| ERR987720 | ERX1068978 | L4 |
| SRR2101123 | SRX1095817 | L4 |
| ERR1023325 | ERX1102197 | L4 |
| ERR1023347 | ERX1102219 | L4 |
| ERR1199098 | ERX1271356 | L4 |
| ERR1199100 | ERX1271358 | L4 |
| ERR1199109 | ERX1271367 | L4 |
| ERR1199124 | ERX1271382 | L4 |
| ERR1199140 | ERX1271398 | L4 |
| ERR2124096 | ERX2180948 | L4 |
| ERR278516 | ERX251849 | L4 |
| ERR386875 | ERX359105 | L4 |
| ERR987705 | ERX1068963 | L4 |
| ERR1023376 | ERX1102248 | L4 |
| ERR278539 | ERX251872 | L4 |
| ERR278608 | ERX251941 | L4 |
| ERR294258 | ERX267591 | L4 |
| ERR386898 | ERX359128 | L4 |
| ERR387023 | ERX359253 | L4 |
| SRR2100924 | SRX1095618 | L4 |
| SRR2100926 | SRX1095620 | L4 |
| SRR2101041 | SRX1095735 | L4 |
| SRR5818632 | SRX2996811 | L4 |
| SRR5818696 | SRX2996747 | L4 |
| ERR046838 | ERX023900 | L4 |
| ERR046839 | ERX023901 | L4 |
| SRR3105801 | SRX1534153 | L4 |
| ERR351912 | ERX324695 | L4 |
| ERR369613 | ERX342322 | L4 |
| ERR369635 | ERX342344 | L4 |
| ERR369643 | ERX342352 | L4 |
| ERR369663 | ERX342372 | L4 |
| ERR369665 | ERX342374 | L4 |
| ERR369700 | ERX342409 | L4 |
| ERR369715 | ERX342424 | L4 |
| ERR369739 | ERX342448 | L4 |
| ERR369750 | ERX342459 | L4 |
| ERR386861 | ERX359091 | L4 |
| ERR551151 | ERX510220 | L4 |
| ERR037533 | ERX1973371 | L4 |
| ERR1035083 | ERX1113881 | L4 |
| ERR1213944 | ERX1286157 | L4 |
| ERR176488 | ERX152634 | L4 |
| ERR387056 | ERX359286 | L4 |
| ERR403404 | ERX369765 | L4 |
| ERR551689 | ERX510758 | L4 |

| | | |
|---|---|---|
| ERR987752 | ERX1069010 | L4 |
| SRR5007176 | SRX2338825 | L4 |
| SRR5007191 | SRX2338840 | L4 |
| ERR1465983 | ERX1536460 | L4 |
| ERR2228926 | ERX2282265 | L4 |
| ERR245771 | ERX220305 | L4 |
| ERR386870 | ERX359100 | L4 |
| ERR551010 | ERX510079 | L4 |
| ERR894411 | ERX973814 | L4 |
| SRR3082143 | SRX1514058 | L4 |
| SRR5153858 | SRX2472020 | L4 |
| SRR6044772 | SRX3192074 | L4 |
| SRR6045453 | SRX3192413 | L4 |
| SRR6067286 | SRX3209766 | L4 |
| ERR1023299 | ERX1102171 | L4 |
| ERR1664664 | ERX1734975 | L4 |
| ERR270631 | ERX244858 | L4 |
| ERR551003 | ERX510072 | L4 |
| ERR551053 | ERX510122 | L4 |
| ERR552439 | ERX511508 | L4 |
| ERR552785 | ERX511854 | L4 |
| ERR553319 | ERX512388 | L4 |
| ERR159958 | ERX135903 | L7 |
| ERR159959 | ERX135904 | L7 |
| ERR181435 | ERX157545 | L7 |
| ERR1971865 | ERX2034751 | L7 |
| ERR400415 | ERX366776 | L7 |
| ERR756344 | ERX700974 | L7 |
| ERR756345 | ERX700975 | L7 |
| ERR756346 | ERX700976 | L7 |
| ERR756347 | ERX700977 | L7 |
| ERR756348 | ERX700978 | L7 |
| ERR234679 | ERX209184 | L5 |
| ERR2704808 | ERX2719151 | L5 |
| ERR2704809 | ERX2719152 | L5 |
| ERR294242 | ERX267575 | L5 |
| ERR294243 | ERX267576 | L5 |
| ERR294244 | ERX267577 | L5 |
| ERR386983 | ERX359213 | L5 |
| ERR386988 | ERX359218 | L5 |
| ERR4162011 | ERX4127340 | L5 |
| SRR2101040 | SRX1095734 | L5 |
| SRR2101063 | SRX1095757 | L5 |
| SRR7496542 | SRX4365927 | L5 |
| ERR1023220 | ERX1102092 | L6 |
| ERR1023230 | ERX1102102 | L6 |

| | | |
|---|---|---|
| ERR1023280 | ERX1102152 | L6 |
| ERR1023290 | ERX1102162 | L6 |
| ERR234106 | ERX208611 | L6 |
| ERR234254 | ERX208759 | L6 |
| ERR351927 | ERX324710 | L6 |
| ERR387025 | ERX359255 | L6 |
| ERR403344 | ERX369705 | L6 |
| ERR551830 | ERX510899 | L6 |
| ERR970410 | ERX1047374 | M. suricattae |
| SRR3500411 | SRX1758447 | M. mungi |
| ERR2659169 | ERX2675547 | M. microti |
| ERR2659170 | ERX2675548 | M. microti |
| ERR2659171 | ERX2675549 | M. microti |
| ERR551111 | ERX510180 | M. microti |
| ERR552037 | ERX511106 | M. microti |
| ERR553376 | ERX512445 | M. microti |
| SRR3647357 | SRX1830303 | M. microti |
| ERR4143897 | ERX4111166 | M. pinipedii |
| ERR4143898 | ERX4111165 | M. pinipedii |
| SRR1239338 | SRX517311 | M. pinipedii |
| SRR7693090 | SRX4552553 | M. pinipedii |
| SRR7693584 | SRX4552651 | M. pinipedii |
| ERR228223 | ERX202882 | M. orygis |
| ERR234675 | ERX209180 | M. orygis |
| ERR234676 | ERX209181 | M. orygis |
| ERR386816 | ERX359046 | M. orygis |
| SRR10251194 | SRX6969200 | M. orygis |
| SRR10251196 | SRX6969198 | M. orygis |
| SRR10251197 | SRX6969197 | M. orygis |
| SRR10251199 | SRX6969195 | M. orygis |
| SRR10251200 | SRX6969194 | M. orygis |
| SRR10251203 | SRX6969191 | M. orygis |
| SRR10321149 | SRX7032003 | M. orygis |
| SRR5642712 | SRX2880898 | M. orygis |
| ERR161048 | ERX136898 | M. bovis |
| ERR228166 | ERX202825 | M. bovis |
| ERR234151 | ERX208656 | M. bovis |
| ERR387001 | ERX359231 | M. bovis |
| ERR046750 | ERX023812 | M. bovis |
| ERR046989 | ERX024051 | M. bovis |
| ERR229952 | ERX204503 | M. bovis |
| ERR552796 | ERX511865 | M. bovis |
| ERR751369 | ERX695051 | M. bovis |
| SRR6045214 | SRX3192142 | M. bovis |
| ERR1462588 | ERX1533066 | M. bovis |

| | | |
|---|---|---|
| ERR1462589 | ERX1533067 | M. bovis |
| ERR1462590 | ERX1533068 | M. bovis |
| ERR1462591 | ERX1533069 | M. bovis |
| ERR1462592 | ERX1533070 | M. bovis |
| ERR1462593 | ERX1533071 | M. bovis |
| ERR1462594 | ERX1533072 | M. bovis |
| ERR1462595 | ERX1533073 | M. bovis |
| ERR1462596 | ERX1533074 | M. bovis |
| ERR1462598 | ERX1533076 | M. bovis |
| ERR751371 | ERX695053 | M. bovis |
| ERR751397 | ERX695079 | M. bovis |
| ERR2659158 | ERX2675536 | dassie |
| ERR713575 | ERX657497 | chimpanzee |
| ERR502505 | ERX467862 | L5 |
| ERR1334049 | ERX1405641 | L5 |
| ERR702413 | ERX646676 | L5 |
| ERR702407 | ERX646670 | L5 |
| ERR1203061 | ERX1275303 | L5 |
| ERR702426 | ERX646689 | L5 |
| ERR502515 | ERX467872 | L5 |
| ERR1082123 | ERX1161713 | L5 |
| ERR1082116 | ERX1161706 | L6 |
| ERR1082136 | ERX1161726 | L6 |
| ERR751306 | ERX694988 | L6 |
| ERR502516 | ERX467873 | L6 |
| ERR502530 | ERX467887 | L6 |
| ERR1215471 | ERX1287684 | L6 |
| ERR1082133 | ERX1161723 | L6 |
| ERR1082141 | ERX1161731 | L6 |
| ERR502533 | ERX467890 | L6 |
| SRR7240037 | SRX4146157 | M. bovis |
| ERR564353 | ERX523378 | M. bovis |
| ERR564390 | ERX523415 | M. bovis |
| SRR7617424 | SRX4482153 | M. bovis |
| SRR1792162 | SRX867824 | M. bovis |
| SRR7240222 | SRX4145972 | M. bovis |
| ERR841835 | ERX921903 | M. bovis |
| SRR7240322 | SRX4145872 | M. bovis |
| SRR7240512 | SRX4146220 | M. bovis |
| SRR1791891 | SRX867553 | M. bovis |
| SRR10828835 | SRX7502208 | L8 |
| ERR4192404 | ERX4153806 | L9 |
| ERR4192405 | ERX4153807 | L9 |
| ERR4192384 | ERX4153786 | L9 |
| ERR4162024 | ERX4127353 | L9 |
